# Predator–prey dynamics of *Vibrio cholerae* on chitin suggest an alternative mode of biofilm formation in marine snow conditions

**DOI:** 10.1093/ismejo/wrag072

**Published:** 2026-03-30

**Authors:** Jacob D Holt, Katherine A Miller, Olivia F Hunter, Emily Zhang, Alexander J Hinbest, Emma Gerace, Rich Olson, Daniel E Kadouri, Carey D Nadell

**Affiliations:** Department of Biological Sciences, Dartmouth, Hanover, NH, 03755, United States; Department of Microbiology and Immunology, Geisel School of Medicine at Dartmouth, Hanover, NH, 03755, United States; Department of Biological Sciences, Dartmouth, Hanover, NH, 03755, United States; Department of Biological Sciences, Dartmouth, Hanover, NH, 03755, United States; Department of Oral Biology, Rutgers School of Dental Medicine, Newark, NJ, 07101, United States; Department of Molecular Biology and Biochemistry, Molecular Biophysics Program, Wesleyan University, Middletown, CT, 06459, United States; Department of Molecular Biology and Biochemistry, Molecular Biophysics Program, Wesleyan University, Middletown, CT, 06459, United States; Department of Molecular Biology and Biochemistry, Molecular Biophysics Program, Wesleyan University, Middletown, CT, 06459, United States; Department of Oral Biology, Rutgers School of Dental Medicine, Newark, NJ, 07101, United States; Department of Biological Sciences, Dartmouth, Hanover, NH, 03755, United States; Department of Microbiology and Immunology, Geisel School of Medicine at Dartmouth, Hanover, NH, 03755, United States

**Keywords:** *Vibrio cholerae*, *Bdellovibrio bacteriovorus*, biofilm, regulation, marine snow, predation, matrix, architecture, resistance, cholera

## Abstract

*Vibrio cholerae* is a ubiquitous marine bacterium that solubilizes and consumes chitin in the marine water column. In both the marine environment and the intestinal tract, *V. cholerae* forms biofilms: how do the diverse surfaces that *V. cholerae* encounters influence its biofilm formation and, in turn, shape its ecological interactions with other microbes? Here, we use the interaction between the predator *Bdellovibrio bacteriovorus* and *V. cholerae* as a model to explore how the environmental chitin substrate alters *V. cholerae* biofilm formation and predator–prey dynamics. We find that glass-bound biofilm growth provides strong protection for *V. cholerae* against predation while also allowing a population of predatory *B. bacteriovorus* to remain in place among prey cells. In contrast, chitin-bound biofilm structure offers less protection against *B. bacteriovorus* predation and does not maintain as stable a population of *B. bacteriovorus*. Using percolation and population dynamics models, we predict that these changes in predator–prey dynamics can be explained largely by alterations in *V. cholerae* biofilm architecture between the two conditions, which changes the fraction of prey available to *B. bacteriovorus*. Using targeted biofilm matrix gene deletions, we confirm this prediction by recapitulating key features of the chitin predator–prey interactions on glass surfaces. Following on this observation, we show that *V. cholerae* biofilms grown on chitin produce much less of the canonical biofilm matrix components and instead rely on other extracellular structures. Overall, our experiments detail how growth substrate can alter biofilm matrix composition and how these changes in biofilm architecture impact higher-order ecological interactions.

## Introduction

*Vibrio cholerae* is the causative agent of cholera and a marine bacterium that forms biofilms on the surface of chitin, where it secretes extracellular enzymes that degrade the insoluble polymer into soluble saccharides [1–5]. Biofilm formation is a distinct physiological state that arises when planktonic cells become attached to a surface and/or to each other; reduce or halt motility; and secrete extracellular matrix composed of proteins, polysaccharides, and extracellular DNA [6–8]. Biofilm formation stabilizes bacterial colonization of nutrient-rich surfaces and in this instance links *V. cholerae* and other chitin-consuming marine bacteria to global carbon and nitrogen cycling [9–13]. Additionally, biofilm formation provides protection against exogenous threats such as antibiotics, desiccation, bacteriophages, host immune systems, competing microbes, and both prokaryotic and eukaryotic predators [14–24]. Though biofilm formation on glass surfaces has been well characterized in protecting *V. cholerae* from invading bacteria, bacteriophages, and predators, it remains unclear how environmental growth substrates of *V. cholerae*, such as chitin, may alter the protection offered by biofilm formation and what implications this may then have for biofilm community dynamics in aquatic environments [15–17, 23].

Recent work has indicated that the ecology and evolution of biofilm-dwelling bacteria are sensitive to the mechanistic details of matrix secretion and biofilm architecture [2, 24–27]. For instance, for *V. cholerae* biofilms grown on glass under continuous media flow, disruption of the matrix protein RbmA leads to modified cell group architecture with greater spacing between cells and high sensitivity to invasion by competing strains, predatory bacteria, and phages [16, 23, 28, 29]. Our goal in this paper was to assess the differences in predator–prey interaction between *Bdellovibrio bacteriovorus* and *V. cholerae* on chitin surfaces in comparison with glass surfaces. We anticipated that microbial predator–prey dynamics might differ in these two conditions, which could then suggest possible changes in *V. cholerae* biofilm matrix physiology on chitin that dictate the extent to which *B. bacteriovorus* can access and kill prey cells. Overall, our goal was to provide a direct illustration of how fundamental ecological dynamics are dictated by the mechanistic details of biofilm production and how, in turn, ecologically motivated experiments for predator–prey interaction can be used to assess differences in underlying biofilm matrix physiology.

To study the effect of chitin-bound growth on biofilm formation and predator–prey dynamics, we implemented *V. cholerae* biofilm growth on chitin particles and exposed them to *B. bacteriovorus*, an obligate predator of Gram-negative bacteria [30]. We also conducted comparison experiments on glass surfaces using one of two different carbon sources in the perfused liquid medium: the soluble disaccharide (GlcNAc)_2_ and the soluble monosaccharide GlcNAc, which are the primary end-products of *Vibrio*-secreted chitinases [4]. We then employed quantitative biofilm architecture measurements, ecological and physical models, and genetic manipulation of matrix production to assess how growth on chitin changes *V. cholerae* biofilm architecture and how these changes, in turn, impact the biofilm population’s interaction with predatory bacteria. Altogether, our findings show that the details of surface association and growth substrate can feed back to biofilm matrix regulation and composition, and these changes, in turn, can cascade to influence ecological interactions on larger spatial scales.

## Materials and methods

### Bacterial strains

The *V. cholerae* strains used in this study were derived from serotype O1 El Tor strain N1691 [31]. The *B. bacteriovorus* strain was derived from 109 J [30]. All mutant derivatives were generated here and previously using standard allelic exchange methods with counter-selection for scarless deletion or integration of reporter constructs or point mutations [2, 5, 16, 23, 32]. Cultures were grown overnight in lysogeny broth (LB, Miller) prior to inoculation into microfluidic devices. Biofilm cultures were grown in M9 minimal media, supplemented with 2 mM MgSO_4_, 100 μM CaCl_2_, and Minimal Essential Medium (MEM) vitamins, with chitin flakes, Chitobiose [(GlcNAc)_2_] at 0.5%, or GlcNAc at 0.5% as the sole carbon source. For a list of strains and materials, see Table S1.

### Microfluidic flow device assembly

Microfluidic chambers used for biofilm culture were made with polydimethylsiloxane (PDMS) using standard soft lithography techniques [33, 34]. PDMS was mixed and then cured on molds of chamber sets, punched with holes for inlet and outlet tubing, and bonded to #1.5 22 mm by 60 mm glass coverslips via plasma cleaning. The microfluidic chambers (7620 μm in length, 1500 μm in width, and 80 μm in height) were designed to hold pieces of chitin flake in place using posts arranged in a “V” shape, with the open end facing upstream into the flow path (SI Fig. S1) [2]. Prior to inoculation with bacteria, sterilized chitin flakes (Sigma) were loaded into the chambers, which were inspected to confirm that pieces of chitin were immobilized by the V-traps for subsequent *V. cholerae* colonization. Constant flow was applied via Harvard Apparatus Pico Plus syringe pumps loaded with 1 ml Brandzig plastic syringes. Syringes had 27-gauge needles affixed to them and fitted with #30 Cole Parmer Polytetrafluoroethylene (PTFE) tubing with an inner diameter of 0.3 mm. Tubing from the syringes was led to the inlets of each chamber, and outlet tubing was fed to a Petri plate to collect waste.

### Biofilm culture conditions

Strains were grown overnight in LB medium at 37°C and shaken at 250 rpm. Overnight cultures were resuspended in M9 minimal media to an O.D._600_ of ~4. For biofilms grown on chitin, molecular-grade chitin flakes were incubated with ethanol for 1 min prior to being washed 5× with phosphate-bufferd saline (PBS) and then resuspended in M9 with no carbon source. These chitin flakes were then flushed into microfluidic chambers to immobilize them in the V-trap sections of the devices (SI Fig. S1) [2]. For biofilms grown on glass with soluble carbon sources, the same microfluidic devices were used, but the disaccharide (GlcNAc)_2_ or the monosaccharide GlcNAc was added to the M9 media at 0.5%. Even though the exact ratio of soluble sugar produced by chitin degradation remains unclear, (GlcNAc)_2_ and GlcNAc are likely end-products of *Vibrio* chitinases [4]. After loading the chambers with chitin flakes, 10 μl of inoculum was transferred into the chambers and allowed to stay in place without flow for 1 h to permit bacterial colonization of the chitin particles. After this colonization step, M9 minimal media were introduced to the chambers at a rate of 0.1 μl/min (corresponding to an average flow velocity of ~14 μm/s) at room temperature (~22°C).

### Biofilm predation assay

After 72 h of biofilm growth, the inlet media was swapped for 2 h to either (i) M9 media containing *B. bacteriovorus* resuspended in M9 media at a concentration of 10^10^ plaque forming units (PFUs) per milliliter or (ii) for control chambers, the same inlet swap procedure was performed but to a media supply that did not contain any *B. bacteriovorus*. Following the 2 h introduction of predator bacteria or sterile control, the media inlet was then swapped back to the original sterile M9 media, and flow was paused for 30 min to allow for adherence of the invading *B. bacteriovorus*. Flow of sterile media was then resumed for the duration of the experiment. Imaging was performed at 4 h postinvasion and at 24 h intervals thereafter. The biovolume of strains, as a proxy for their population size, was measured by imaging *z*-stacks of multiple 212 × 212 μm fields of view within each replicate chamber.

### Plaque assay

Standard double-layer agar plates were used to plaque *B. bacteriovorus* on *V. cholerae* N16961 wild type (WT) and *∆vpsL* prey cells. *Vibrio cholerae* overnights were grown in LB before being resuspended in 4-(2-hydroxyethyl)-1-piperazineethanesulfonic acid (HEPES) buffer at an O.D._600_ of 4. Next, 100 μl of a 10^9^ stock of *B. bacteriovorus* was mixed with 400 μl of *V. cholerae* overnight and 2.5 ml of liquified dilute nutrient broth soft agar supplemented with 2 mM calcium chloride and 3 mM magnesium chloride. This mixture was then spread onto dilute nutrient broth hard agar plates supplemented with 2 mM calcium chloride and 3 mM magnesium chloride. *Bdellovibrio bacteriovorus* plaques were counted after 7 d of incubation at room temperature (~22°C).

### Predation localization

To quantify the probability of predation with respect to biofilm local cell packing, the biofilm predation assay was repeated as previously described, but imaging was performed 2 h after predator introduction. Given that the latent period of *B. bacteriovorus* is as short as 3–4 h, this 2 h time point ensured that images were collected prior to *V. cholerae* predation-driven cell lysis [35–37]. We then aggregated our image data from the chitin, (GlcNAc)_2_, and GlcNAc treatments such that there was a uniform distribution of local cell packing values before calculating the probability of predation with respect to local biofilm structure.

### Percolation model of predation

To model the probability of predation events with respect to local cell packing, we used a percolation model with a spherical geometry [38]. A 3D sphere with a radius of 12 voxels was randomly populated with site occupation probabilities ranging from 0 to 1. For each site occupation probability, 400 spherical grids were generated. A search for a connected path from the exterior of the sphere to the center through unoccupied grid points was performed on each grid. The center of the sphere was defined as a 4 × 4 × 4 voxel cube. Setting voxel dimension to 0.5 μm^3^, approximately the size of a *B. bacteriovorus* cell, these dimensions approximate the BiofilmQ pseudo-cell cube object and cell packing quantification. If a path from outside to the center of the 3D grid was found, that simulation was counted as a successful predation event. If a path was not found, that simulation was counted as a failed predation event. For a graphical illustration in two dimensions (a circular grid) detailing how this model relates to our experimental data, see SI Fig. S4. SI Table S2 provides a list of parameters and units.

### Predator–prey model

To model predator–prey dynamics, we used a classical Lotka–Volterra model [39]. We monitor a prey species, *N* (μm^3^), that grows in a density-dependent manner: $\frac{dN}{dt}= rN\left(\frac{K-N}{K}\right)$, where *r* is the maximal growth rate (d^−1^), and *K* is the carrying capacity (μm^3^). We then introduce a predator, *P* (μm^3^), that has a loss rate *d* (d^−1^) and attacks the prey species at rate *α* (μm^−3^ d^−1^), generating *B. bacteriovorus* cells that are actively digesting infected prey, termed bdelloplasts (*B*, um^3^). Bdelloplasts mature into new predators with conversion efficiency *b* (unitless) and rate *k_p_* (d^−1^), giving the set of equations:


$$ \frac{dN}{dt}= rN\left(\frac{K-N}{K}\right)- aNP, $$

$$ \frac{dP}{dt}={k}_p bB- aPN- dP ,$$

$$ \frac{dB}{dt}= aPN-{k}_pB $$

To simulate the effect of biofilm local cell packing blocking predator access, we added an additional interaction term, *v*, giving:


$$ \frac{dN}{dd}= rN\left(\frac{K-N}{K}\right)- avNP, $$

$$ \frac{dP}{dt}={k}_p bB- avPN- dP, $$

$$ \frac{dB}{dt}= avPN-{k}_pB. $$

For a list of parameters, see SI Table S3.

### Matrix component staining assay

RbmA, Bap1, and RbmC were engineered to carry a C-terminal 3xFLAG epitope as previously described [16, 25, 40]. A Cy3-conjugated anti-FLAG monoclonal antibody was then introduced into the culture medium for the duration of the experiment at 1 μg/ml. To stain Vibrio exopolysaccharide (VPS), Alexa fluor 488 was fused to Bap1 and purified protein was spiked into the chambers at a concentration of 1 μM and allowed to incubate without flow for 0.5 h [41]. After 0.5 h, sterile media flow was resumed to wash out unbound stain. Biofilms were grown as described in the Biofilm Culture Conditions section. For dual carbon source experiments, the soluble carbon source concentration was decreased to 0.005% and the BiofilmQ colony separation function was used to separate biofilms formed on chitin from biofilms formed off of chitin when the two cooccurred within the same field of view as distinct biofilm formations. Stain signal was quantified within a 0.75 μm shell surrounding segmented *V. cholerae* biovolume.

### Biofilm competition assay

The two strains in each competition assay were inoculated into microfluidic devices at O.D._600_ ~ 4.0 at initial ratios ranging from 100:1 to 1:100 (Strain1:Strain 2); initiating these experiments at a range of ratios allows for identification of frequency dependence in the competition outcomes. To establish the exact initial ratio of strains that colonized the chambers, each chamber was imaged 1 h postinoculation via confocal microscopy. After 5 d of biofilm growth, the relative abundances of the competitors were again measured via confocal microscopy. Biofilms were maintained as described in the biofilm culture conditions section.

### Fluorescence microscopy

All imaging was performed using a Zeiss 880 line-scanning confocal microscope with a 40×/1.2 N.A. water objective. The GFP protein expressed constitutively by *B. bacteriovorus* was excited with a 488 nm laser line. The mKO-κ protein that *V. cholerae* expresses constitutively was excited with a 543 nm laser line. The mKate2 protein that *V. cholerae* expresses constitutively was excited with a 594 nm laser line. The Cy3 Anti-FLAG was excited with a 543 nm laser line. The Bap1-Alexa488 fusion was excited with a 488 nm laser line. The chitin, which is auto-fluorescent, was excited with a 405 nm laser line. All representative images were processed by constrained iterative deconvolution in ZEN Blue.

### Image analysis

CZI image files were converted to TIFF format prior to being loaded into either a custom Python script or BiofilmQ, which was run using MATLAB [42]. Thresholding was done using Otsu’s method with a manual sensitivity adjustment [43]. Cube side lengths were set to 2.3 μm, giving cubes that can maximally hold ~12 individual cells. For spatially resolved analyses, the thresholded 3D bacterial populations were segmented into a cubic grid for quantification of parameters at a pseudo-cell resolution unless otherwise noted. Calculated parameters were then exported from BiofilmQ as .mat files and loaded into Python, where SciPy, seaborn, Pandas, NumPy, and Matplotlib were used for running statistical tests and figure generation [44–51]. For calculating the cell-packing cube-based parameter that requires a range, we chose 6 μm as the range over which to measure (see main text, SI Fig. S3, and previous publication [16]). To calculate fluorescence intensity within a range of biovolume, we chose 0.75 μm as the range over which to measure. To account for cubes containing varying amounts of biovolume, cube-based histograms are weighted by the volume contained within each cube. A custom Python script was used to correlate the VPS and RbmA stains. Operation of BiofilmQ and its array of analytical methods is described in extensive detail in Hartmann *et al.* [52]. 3D renderings were generated in either Zen Blue or Paraview. All custom scripts are publicly available on GitHub.

### Replication and statistics

At least three biological replicates, each defined as the average of technical replicate image stacks within a single flow chamber, were collected for each experiment. For biovolume measurements, multiple regions of 212 × 212 μm were sampled as technical replicates within a chamber and averaged for each biological replicate. *Z*-stacks for all experiments were collected at a height of 15 μm due to aberration caused by the chitin at greater imaging depths. For all time-resolved data, the error bars correspond to one standard error. For box and whisker plots, the orange bar denotes the median, the box denotes Q1 and Q3, and the whiskers denote Q1 and Q3 + 1.5 times the interquartile range. The Python scripts and corresponding datasheets used to run models, generate figures, and perform statistical analysis are publicly available on GitHub.

## Results

### *V. cholerae* biofilms formed on chitin are more susceptible to predation by *B. bacteriovorus* than biofilms formed on glass

To observe live-cell population dynamics in biofilms under fluid flow, we used strains of *V. cholerae* and *B. bacteriovorus* that had been engineered to constitutively express the fluorescent proteins mKate2 and GFP, respectively, allowing them to be distinguished by fluorescence microscopy. *Vibrio cholerae* was inoculated into microfluidic devices composed of PDMS chambers bonded to glass coverslips; the chambers contained V-shaped traps that were preloaded with chitin flakes to which *V. cholerae* cells could adhere (SI Fig. 1). After a 1-h attachment period, *V. cholerae* was incubated under a continuous flow of M9 minimal medium—with no carbon source (for experiments in which biofilms grew on chitin), (GlcNAc)_2_, or GlcNAc—at 0.1 μl/min (average flow velocity ~ 14 μm/s). After 72 h of *V. cholerae* biofilm formation, *B. bacteriovorus* was inoculated into the existing *V. cholerae* biofilm chambers via a tubing swap to media containing 10^10^ PFU/ml of *B. bacteriovorus*. After a 2 h invasion period, the tubing was swapped back to the original sterile medium, and imaging was performed 2 h later and every 24 h thereafter until the end of the experiment. To isolate the effect of predation on *V. cholerae* in the three different culture conditions explored here, we ran control experiments in which inoculation and growth conditions were the same as those described above, including the tubing swap at 72 h, but sterile media rather than *B. bacteriovorus* was added to the chambers. We note here also that our focus is on the within-biofilm population dynamics of *V. cholerae* and *B. bacteriovorus*; thus, we are concentrating specifically on the abundances and locations of the two species in direct association with chitin and each other.

**Figure 1:**
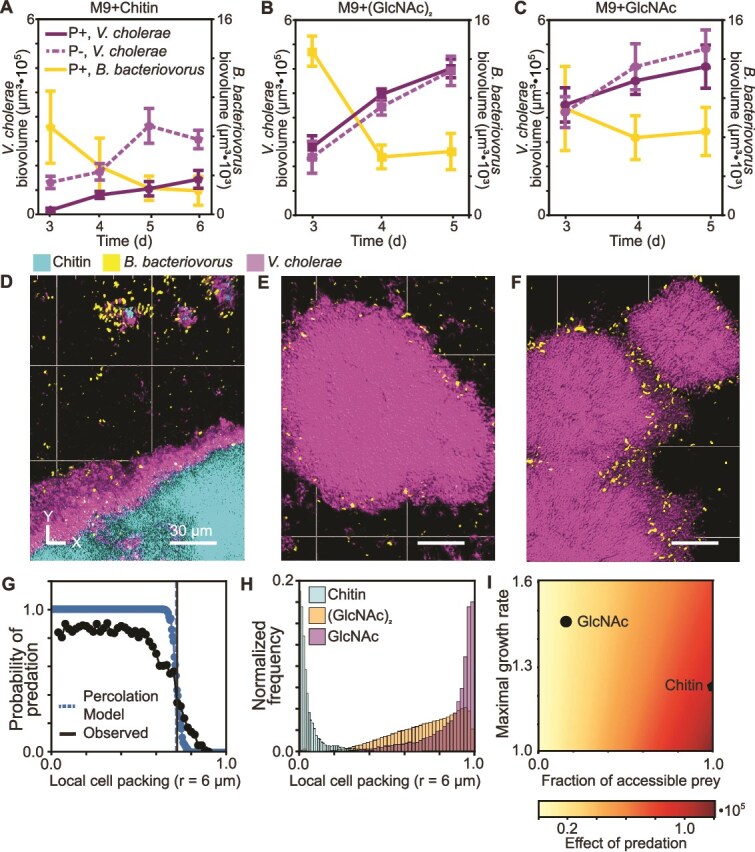
*V. cholerae* N16961 biofilm populations on chitin are more susceptible to *B. bacteriovorus* predation than biofilms formed on glass with GlcNAc or (GlacNAc)_2_ as the carbon source. (A) Population dynamics of *V. cholerae* biofilms formed on chitin with or without *B. bacteriovorus* introduced after 3 d of growth (P+: predator added; P−: control runs with no predator addition). (B) Population dynamics of *V. cholerae* biofilms formed on glass with (GlcNAc)_2_ in the medium, with or without the addition of *B. bacteriovorus*. (C) Population dynamics of *V. cholerae* biofilms formed on glass with GlcNAc in the medium, with or without the addition of *B. bacteriovorus*. (D) Representative 3D projection of a *V. cholerae* biofilm on chitin, following addition of *B. bacteriovorus*. (E) Representative 3D projection of a (GlcNAc)_2_ supplied *V. cholerae* biofilm on glass, following addition of *B. bacteriovorus*. (F) Representative 3D projection of a GlcNAc-supplied *V. cholerae* biofilm on glass, following addition of *B. bacteriovorus*. For all image panels, *V. cholerae* is shown in purple, *B. bacteriovorus* is shown in yellow, and chitin is shown in cyan. (G) Probability of predation as a function of local cell packing plotted from experimental data and from simulation results. The dashed, vertical lines show the point at which the median value becomes zero. (H) Histograms of WT *V. cholerae* biofilm local cell packing across three media conditions: M9 + chitin, M9 + (GlcNAc)_2_, and M9 + GlcNAc. (I) Heatmap showing how the effect of the predator on prey abundance, defined as the time-averaged difference between the predator-treated and -untreated prey populations, changes as the prey maximal growth rate is varied from 0.5 to 1.5 and the fraction of accessible prey is varied from 0 to 1. The chitin and GlcNAc model fits are mapped to the heatmaps as a pentagon and a circle.

When grown in M9 minimal media with chitin as the sole carbon source, a significant decrease in the biovolume of *B. bacteriovorus*–exposed biofilms was observed relative to control chambers that received a tubing swap with sterile media (Fig. 1A, SI Fig. S2A). We next sought to determine if *V. cholerae* biofilms grown on chitin are more or less susceptible to predation by *B. bacteriovorus* relative to biofilms grown on glass with nutrients supplied in the surrounding liquid; to do this, we performed experiments in which *V. cholerae* was grown on the glass bottom of the chambers with either GlcNAc or (GlcNAc)_2_ (0.5%) as the sole carbon source in the liquid media [53]. In contrast to the chitin condition, when *V. cholerae* was grown in M9 media on glass with either GlcNAc or (GlcNAc)_2_, its biofilms were much more resilient to *B. bacteriovorus* exposure. Predation events could be observed along the biofilm periphery, and *B. bacteriovorus* remained associated with the host biofilm, but there was minimal net effect of predation on *V. cholerae* population dynamics (Fig. 1B and C SI Fig. S2B and C).

From our initial set of experiments, we noted three main features distinguishing *V. cholerae* biofilms formed on chitin particles versus those grown on glass with GlcNAc or (GlcNAc)_2_ in the media. First, at the timepoint of predator addition (3 d), the total abundance of the *V. cholerae* growing on chitin was lower than for biofilms on glass with growth substrate in the influent media (Fig. 1A–C, SI Fig. S2, SI Fig. S3A). Second, following the introduction of *B. bacteriovorus* and initial bouts of predation, the total abundance of *B. bacteriovorus* remaining biofilm-associated decreased continuously within biofilms grown on chitin, while *B. bacteriovorus* abundance stabilized within biofilms grown in GlcNAc or (GlcNAc)_2_ (Fig. 1A–C, SI Fig. S3B). Third, *V. cholerae* biofilms grown on chitin exhibited lower internal cell packing and allowed *B. bacteriovorus* to permeate most of their interior volume, while biofilms grown on glass were more densely packed and confined *B. bacteriovorus* to the outside of cell groups (Fig. 1D–F, SI Fig. S2, SI Fig. S3C). The third observation recapitulates our prior work, which found a consistent, negative relationship between the volume fraction occupied by prey bacterial cells and the likelihood of predation by *B. bacteriovorus* [16, 27].

We assessed whether a simple percolation model that considers only the occupation of space by prey cells could predict the likelihood that a focal prey cell could be reached by *B. bacteriovorus*. Briefly, this model considers a 3D spherical grid of voxels as discrete sites of potential prey cell occupancy. Per-site prey occupation probability ranged from 0 to 1 in different simulation runs. For a given run, the model assesses whether a continuously open path exists from the exterior of the sphere to an interior cubic core. This modeling geometry and approach were motivated by the cell packing quantification for experimental data, which measures the bacteria-occupied volume fraction of space within a radius surrounding a central focal pseudo-cell cube (SI Fig. S4). The percolation model gave a good qualitative match to measurements of predation probability as a function of nearby cell packing (Fig. 1G). This result, in turn, suggested that the difference in predator–prey population dynamics between biofilms grown on chitin versus glass could be largely due to differences in prey cell accessibility based on their local cell packing within the biofilm population. To test this possibility, we used a modified Lotka–Volterra model to ask whether the observed difference in cell packing between *V. cholerae* biofilms grown on chitin versus glass (Fig. 1H)—and the resulting predicted difference in predator exposure—could explain the observed difference in predator–prey population dynamics between the different growth conditions. The model was parameterized using monoculture biofilm data (SI Fig. S5), and it produced a partial match to the predator–prey coculture experiments, connecting the difference in cell packing observed between the chitin- and glass-grown biofilm conditions and the observed impact of *B. bacteriovorus* exposure on prey abundance over time (Fig. 1I).

Our model did not capture the population dynamics of *B. bacteriovorus*; we were not able to find a case in which the predator maintained stable positive abundance while having a lower effect on prey abundance (as observed on glass relative to chitin). Instead, we observed a rapid decline of predator abundance in all model cases (SI Fig. S6A and B). After further exploring the model to understand the source of this discrepancy, we determined that to see increased predator abundance as the predator’s available prey pool decreases, there must be a compensatory effect by which either the predator’s loss rate decreases with decreasing prey accessibility, or the predator’s prey conversion efficiency increases with decreasing prey accessibility (SI Fig. S6C–E). This pointed toward an additional subtlety in the experiments, beyond simply where predators and prey cells are located, which we explore further below. Altogether, the results suggest fundamental differences in *V. cholerae* biofilm structure on chitin versus on glass under flow of GlcNAc or (GlcNAc)_2_. In the following section, we manipulate *V. cholerae* matrix composition on glass surfaces to test the hypothesis that relatively simple changes in matrix could account for the major differences in predator–prey interaction on chitin versus glass that we have documented thus far.

### Partially matrix-deficient biofilms formed on glass recapitulate key features of *B. bacteriovorus*–*V. cholerae* interaction on chitin

**Figure 2:**
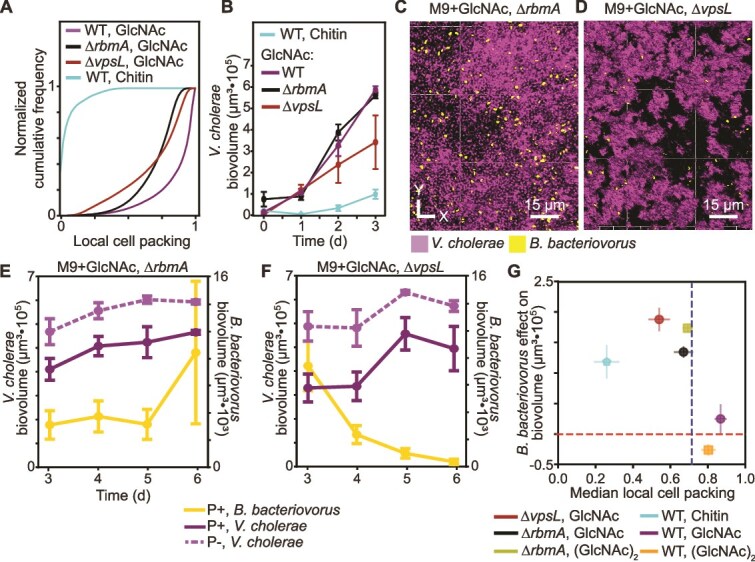
Abolishing *Vibrio* exopolysaccharide (VPS) or RbmA secretion during biofilm formation on glass recapitulates features of predator–prey dynamics on chitin. (A) Cumulative frequency distribution of local cell packing for N16961 WT grown on chitin, WT grown in GlcNAc, ΔvpsL grown in GlcNAc, and ΔrbmA grown in GlcNAc. (B) Biofilm growth curves for WT grown on chitin, WT grown in GlcNAc, ΔvpsL grown in GlcNAc, and ΔrbmA grown in GlcNAc. (C) Representative image of a ΔrbmA biofilm during predation by *B. bacteriovorus*. (D) Representative image of a ΔvpsL biofilm during predation by *B. bacteriovorus*. *Vibrio cholerae* is shown in purple, and *B. bacteriovorus* is shown in yellow. (E) Population dynamics of ΔrbmA *V. cholerae* biofilms formed on chitin with *B. bacteriovorus* added after 3 d of growth (P+, solid line), or without the addition of *B. bacteriovorus* (P−, dashed line). (F) Population dynamics of ΔvpsL *V. cholerae* biofilms formed on chitin with *B. bacteriovorus* added after 3 d of growth, or without the addition of *B. bacteriovorus*. (G) The effect of *B. bacteriovorus* on *V. cholerae* biofilm biovolume plotted against the median monoculture cell packing for five conditions: WT grown on chitin shown in cyan, WT grown in GlcNAc shown in purple, WT grown in (GlcNAc)_2_ shown in orange, ΔvpsL grown in GlcNAc shown in red, and ΔrbmA grown in GlcNAc shown in black. The horizontal red dashed line denotes zero net effect of predation on *V. cholerae* biomass accumulation. The critical threshold, *p*_c_, predicted by the percolation model in Fig. 1G is shown as a vertical, dark-blue dashed line_._

To further define the relationship between matrix composition, cell arrangement, and predator–prey population dynamics, we performed experiments using strains harboring deletions of key extracellular matrix components grown on glass, focusing on the matrix protein RbmA and the core VPS. *Vibrio cholerae* produces at least three other matrix proteins, including RbmC, Bap1, and GluP [54, 55]. However, we opted specifically for RbmA manipulation because it is the only known matrix component that can be singly removed to modulate cell packing density. RbmA is a secreted protein that interacts with the *V. cholerae* cell surface and VPS to control how closely cells are arranged together; deletion of *rbmA* does not eliminate biofilm production but does substantially reduce the density of cells within the surface-bound population (Fig. 2A and B, SI Fig. S7A) [8, 28, 40, 56, 57].

Repeating the predation experiments with biofilms formed by the *∆rbmA* strain, we recapitulated our prior result that *B. bacteriovorus* permeates through most of the biofilm, homogeneously spreading out within the host population [16, 27]. Additionally, as in the chitin case, we observed a reduction in *V. cholerae* abundance compared to a no-predator control (Fig. 2E, SI Fig. S7). However, unlike in the chitin case, *B. bacteriovorus* is retained within *∆rbmA* biofilms at an abundance similar to parental strain biofilms grown on glass (Fig. 1B and C, Fig. 2E, SI Fig. S7). Thus, removal of RbmA from biofilms on glass recapitulates the increased predator susceptibility of biofilms formed on chitin but could not account for the gradual decrease in predator abundance observed in the chitin experiments. This result motivated us to turn to another biofilm matrix component that also results in decreased local cell packing on glass, VPS (Fig. 2A).

To characterize the interaction of *B. bacteriovorus* with *V. cholerae* biofilms that do not contain VPS, we took advantage of a previously characterized *vpsL* deletion mutant. Although the exact function of VpsL is unknown, it is predicted to be a key component of the matrix exopolysaccharide secretion machinery that localizes to the cell inner membrane, and mutants lacking VpsL do not secrete VPS [57, 58]. Because the matrix proteins RbmA, RbmC, and Bap1 all interact with VPS, biofilms lacking VPS are thought not to retain the primary matrix proteins, either. Consequently, *∆vpsL* mutants either fail to produce 3D biofilms or form irregularly shaped biofilms with varying degrees of 3D structure, dependent on media conditions (SI Fig. S8) [2, 41, 57, 59–63]. In M9 + GlcNAc, *V. cholerae ∆vpsL* produces irregularly shaped 3D biofilm clusters with cell packing profiles that are reduced compared to VPS-producing cell groups (Fig. 2A, B, and  D).

Repeating the predation experiment with *V. cholerae ΔvpsL* grown on glass with GlcNAc in the medium flow-through, we observed *B. bacteriovorus* initially permeating most of the *V. cholerae* population and a significant decline in *V. cholerae* abundance compared to a no-predator control (Fig. 2D and F, SI Fig. S7). Even though *V. cholerae* biofilm susceptibility to predation is similar between the *ΔvpsL* and the *∆rbmA* mutants under these culture conditions, the effects of each deletion mutant on *B. bacteriovorus* population dynamics are different. Though they are able to attack and consume prey cells within 24 h of being introduced and can efficiently produce plaques on *∆vpsL* prey cells relative to WT prey cells, *B. bacteriovorus* did not persist within or along the periphery of *∆vpsL* biofilms in the days following (Fig. 2F, SI Fig. S7). Altogether these results indicated that the ability of *B. bacteriovorus* to be maintained on *V. cholerae* biofilms grown on glass substrate is dependent on the presence of VPS and potentially its interactions with other matrix components, and not solely on the abundance of accessible prey.

So far, we have confirmed that, regardless of underlying surface, lowered local cell packing results in an increased susceptibility to predation by *B. bacteriovorus* (Fig. 2G). However, only the inhibition of VPS secretion is sufficient to recapitulate both the increased susceptibility to predation and the gradual decrease in predator abundance observed previously in the chitin experiments. These results suggest that biofilms formed on chitin may not be producing VPS or may produce it in much lower quantities.

### Biofilms formed on chitin have altered matrix configuration relative to biofilms formed on glass

**Figure 3:**
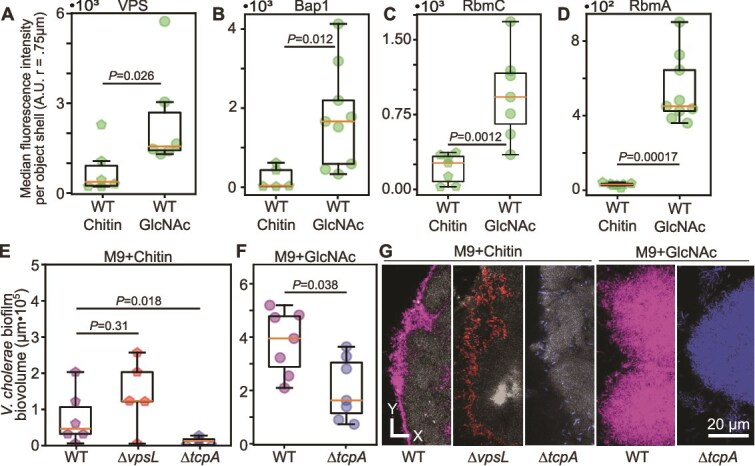
Growth on chitin leads to a shift in biofilm matrix composition. **(**A) VPS, quantified by Bap1-VPS binding domain labeling, is produced significantly less in biofilms on chitin relative to those on glass with GlcNAc in the surrounding medium (Mann–Whitney *U* test, *n* = 6, *P* = .026). (B) Bap1, quantified by immunostaining, is produced significantly less in biofilms on chitin relative to those on glass with GlcNAc (Mann–Whitney *U* test, *n* = 5–9, *P* = .012). (C) RbmC, quantified by immunostaining, is produced significantly less in biofilms on chitin relative to those on glass with GlcNAc (Mann–Whitney *U* test, *n* = 7, *P* = .0012). (D) RbmA, quantified by immunostaining, is produced significantly less in biofilms on chitin relative to those on glass with GlcNAc (Mann–Whitney *U* test, *n* = 7–9, *P* = .00017). (E) Biofilm volume at 3 d of *V. cholerae* WT, *∆vpsL*, and *∆tcpA* grown on chitin as the sole carbon source. There is a significant difference between WT and *∆tcpA* (Mann–Whitney *U* test, *n* = 6–7, *P* = .018) and no significant difference between WT and *∆vpsL* (Mann–Whitney *U* test, *n* = 5–6, *P* = .31). WT data in this panel are replotted from Fig. 2. (F) Biofilm volume at 3 d of *V. cholerae* WT and *∆tcpA* grown on glass with GlcNAc as the sole carbon source (Mann–Whitney *U* test, *n* = 7, *P* = .038). (G) Representative images of WT, *∆vpsL*, and *∆tcpA* growing on chitin (left-most three images) and GlcNAc (right-most two images). WT is shown in purple, *∆vpsL* is shown in red, *∆tcpA* is shown in blue, and chitin is shown in gray.

Our observations thus far prompted us to ask whether VPS and the other primary matrix components—RbmA, Bap1, and RbmC—were being produced in lower quantities when *V. cholerae* is attached to chitin [25, 27]. To assess production of VPS, we used a previously described approach in which the β-propeller domain of Bap1, which binds to VPS, is conjugated to a fluorophore to serve as a VPS-specific fluorescent stain [41]. We observed diffuse and bright stain signal throughout biofilms grown in GlcNAc on glass, while biofilms formed on chitin showed little to no signal (Fig. 3A, SI Fig. S9A). Using a previously described immunostaining approach for the secreted proteins RbmA, Bap1, and RbmC, we also observed significantly reduced matrix protein production within biofilms grown on chitin relative to those formed on glass with GlcNAc in the surrounding medium (Fig. 3B–D, SI Fig. S9B–D) [16, 23, 25, 40].

We inferred that for our strains and culture conditions, VPS-dependent biofilm formation as a whole is activated to a lower degree when *V. cholerae* is growing on chitin. To confirm that this result was not an effect of delayed biofilm maturation, we performed a dual carbon source experiment in which solubilized GlcNAc (0.005%) was added to chambers that also contained chitin flakes. This setup was designed to allow cells to grow on chitin or on glass, with GlcNAc available in the influent medium, and thus to allow us to infer whether underlying surface or solubilized GlcNAc availability was the primary determinant of VPS production. In this dual carbon source experiment, we found that cells in association with the chitin surface displayed substantially decreased RbmA stain signal relative to cells growing off of the chitin surface (SI Fig. S10).

If VPS and its associated matrix proteins indeed contribute little to *V. cholerae* biofilm growth on chitin, we predicted that a *∆vpsL* mutant, which cannot secrete VPS, should grow similarly to its isogenic parental strain on chitin, which we confirmed (Fig. 3E and G). Further supporting our interpretation of decreased VPS production on chitin, in two-strain competition experiments performed on chitin or on glass with GlcNAc, the *∆vpsL* mutant had a higher competitive fitness against its parental strain on chitin relative to the glass and GlcNAc growth condition (SI Fig. S11A and B). These outcomes, together with the matrix component staining results above, suggest that one or more underlying surface properties are relayed intracellularly and play a major role in the regulation of *V. cholerae* matrix production. This then prompted us to consider other possibilities for cellular structures holding cells to each other in association with the chitin surface in these experiments.

Prior studies have suggested that the Type 4 pili of *V. cholerae* can be used for aggregation, as well as biofilm formation in some contexts [60, 64–69]. Specifically, in our strain background, N16961, the Toxin Coregulated Pilus (TCP) has been implicated as a necessary structure for biofilm maturation on chitin derived from squid pens [70]. To test if the TCP is related to biofilm formation in our experiments, we constructed a *∆tcpA* deletion mutant and its chitin-bound biofilm growth.

After 3 d of incubation on chitin, *∆tcpA* biofilms exhibited significantly reduced size compared to the WT control (Fig. 3E and G). For comparison, we repeated this experiment using glass as the substratum for biofilm growth and GlcNac as the sole carbon source in the surrounding medium. In this context, the *∆tcpA* strain also showed reduced biomass compared to WT, but it was able to produce microcolony structures indicative of well-studied VPS-dependent biofilm formation (Fig. 3F and G). The *∆tcpA* mutant also had a competitive disadvantage against WT in biofilms on glass with GlcNAc and on chitin, further supporting a role for TCP in biofilm integrity (SI Fig. S11C and D). These results align with prior work and suggest that biofilm formation on chitin is dependent on production of one or more pili; the results are also consistent with reports that *tcpA* expression levels are elevated in biofilms formed both on and off of chitin [64, 70, 71].

### Hyperactive diguanylate cyclase mutant forms biofilms on chitin that are protected from *B. bacteriovorus* predation

**Figure 4:**
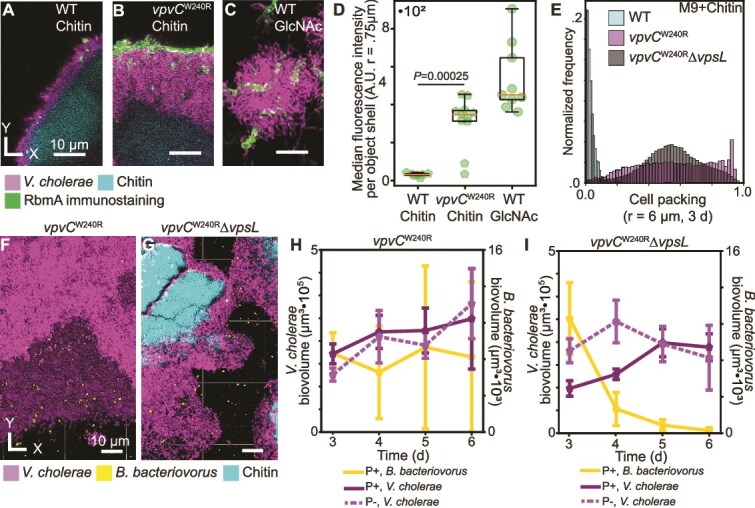
A hyperactive diguanylate cyclase mutant, *vpvC*^W240R^, forces VPS-dependent biofilm formation on chitin, resulting in similar predator–prey dynamics to those observed in biofilms on glass. (A) Representative image of N16961 WT biofilm on chitin after addition of RbmA immunostain. (B) Representative image of *vpvC*^W240R^ biofilm grown on chitin after addition of RbmA immunostain. (C) Representative image of WT biofilm grown on glass with GlcNAc after addition of RbmA immunostain. In (A–C), *V. cholerae* is shown in purple, RbmA immunostain signal is shown in green, and chitin is shown in cyan. (D) Box and whisker plot illustrating a significant increase in RbmA immunostaining between *vpvC*^W240R^ and WT on chitin (Mann–Whitney *U* test, *n* = 7–11, *P* = .00025, WT data replotted from Fig. 3). (E) Cell packing frequency distributions of WT, *vpvC*^W240R^, and *vpvC^W240R^ΔvpsL* biofilms grown on chitin. (F) Representative 3D rendering of a *vpvC*^W240R^ biofilm being predated by *B. bacteriovorus*. (G) Representative 3D rendering of a *vpvC*^W240R^*ΔvpsL* biofilm being predated by *B. bacteriovorus*. (F, G) *V. cholerae* is shown in purple, *B. bacteriovorus* is shown in yellow, and chitin is shown in cyan. (H) Population dynamics of *vpvC*^W240R^ with (P+, solid line) and without (P−, dashed line) *B. bacteriovorus* predation. (I) Population dynamics of *vpvC*^W240^*ΔvpsL* with and without predation.

If our inference that VPS-dependent biofilm growth is reduced on chitin in favor of other mechanisms that could hold cells together in proximity to the surface, we predict that increasing VPS-dependent biofilm growth in the chitin environment via genetic modification will restore a similar degree of prey protection and predator abundance as seen in biofilms grown on glass. To test this possibility, we used a strain of *V. cholerae* harboring a point mutation, *vpvC*^W240R^, previously characterized as elevating intracellular c-di-GMP concentration and leading to constitutive production of VPS and the matrix proteins RbmA, RbmC, and Bap1 [16, 72]. We confirmed this strain behaves as expected in our culture conditions using RbmA immunostaining (Fig. 4A–D, SI Fig. S11). RbmA immunostaining corresponds strongly with VPS staining, making it suitable as a general matrix stain in *V. cholerae* biofilms (SI Fig. S12) [16].

*vpvC* ^W240R^ biofilms grown on chitin have substantially increased cell packing relative to a WT control. Because the *vpvC*^W240R^ mutation increases c-di-GMP levels, which can influence the expression of many loci, it was also necessary to show that changes in *vpvC*^W240R^ biofilm morphology on chitin were specifically due to the VPS-dependent matrix, rather than other factors. We performed this check by making a *vpsL* deletion in the *vpvC*^W240R^ background, which resulted in a strain with similar biofilm accumulation after 3 d, but decreased cell packing relative to *vpvC*^W240R^ when grown on chitin (SI Fig. S13A). The double mutant strain did still produce more tightly packed biofilms than the WT on chitin, however, suggesting moderate roles for other factors altered by elevated c-di-GMP pools (Fig. 4E) that we do not explore further here. Invading *B. bacteriovorus* into mature *vpvC*^W240R^ biofilms formed on chitin, we observed *B. bacteriovorus* localization to the biofilm exterior, a negligible effect of *B. bacteriovorus* on *vpvC*^W240R^ biofilm abundance over time, and increased retention of *B. bacteriovorus*, consistent with our predictions and data from the previous sections (Fig. 4F and H, SI Fig. S13). Consistent with the prior experiments with biofilms lacking VPS, *B. bacteriovorus* has a measurable effect on the abundance of *vpvC*^W240R^Δ*vpsL* biofilms at early timepoints, but as *B. bacteriovorus* is lost over time, the effect of the predator is no longer observed (Fig. 4G and I, SI Fig. S13). Together, these results confirm that the altered predator–prey dynamics on chitin can be largely explained by a substantial decrease in VPS production.

## Discussion

Pathogenic *V. cholerae*’s life cycle involves shifts between the human host and the marine water column [3]. In both host digestive tracts and marine conditions, *V. cholerae* forms biofilms to sequester space and growth resources and to survive contact with a myriad of external threats including elements of the host immune system, bacteriophages, predators, and competing bacterial strains and species [2, 16, 23, 60]. Although *V. cholerae* is capable of forming pilus-dependent aggregates, it is generally thought to be VPS and related matrix proteins that contribute to robust *V. cholerae* biofilm formation [8, 57, 60, 64–68, 70, 73]. How different growth conditions may alter the extent of matrix production and how these changes cascade to changes in ecological interaction with other microbes remain fruitful areas of work.

Here, we used the predatory bacterium *B. bacteriovorus* in conjunction with *V. cholerae* biofilm formation on chitin and glass substrates to explore how different environmental contexts influence *V. cholerae* biofilm architecture and predator–prey ecology [4, 53]. We first showed that biofilm growth on chitin increases *V. cholerae*’s susceptibility to predation by *B. bacteriovorus* while simultaneously decreasing the abundance of *B. bacteriovorus* from the system. We next used matrix deletion mutants cultivated on glass to recapitulate these key observations from the chitin experiments, and we demonstrated that VPS and matrix protein production are substantially reduced on chitin relative to glass surfaces. Furthermore, the *V. cholerae* TCP appears to play a role in biofilm integrity on chitin surfaces, and a more modest role on glass surfaces. Finally, we showed that if VPS and related matrix protein production are induced on glass via genetic modification, biofilms regain key features of predator–prey interactions and biofilm architecture normally seen during glass cultivation.

Our finding that chitin-adhered biofilms are more susceptible to predation than VPS-dependent biofilms grown on glass is suggestive of a tradeoff that *V. cholerae* likely contends with in the marine environment. Pieces of chitin detritus offer a finite source of carbon and nitrogen, and producing large and densely packed biofilms, such as those made by *vpvC*^W240R^, can offer competitive benefits and protection from exogenous threats. However, biofilms produced by matrix hypersecretion have been shown to incur a cost in terms of dispersal and downstream colonization ability [74, 75]. This suggests a tradeoff that could provide an evolutionary rationale for the existence of *V. cholerae* variants from the marine environment that differ in their propensity to produce VPS and its associated matrix proteins [72]. In the future, expanded study of *V. cholerae* biofilm growth and dispersal for recolonization of new particles, including how predatory species such as *B. bacteriovorus* interact with their prey in the planktonic phase in transit from one particle to another, will help to resolve this question.

The finding that VPS, RbmA, Bap1, and RbmC are produced to a lesser degree on chitin relative to GlcNAc-grown biofilms corresponds with prior reports of the Toxin-Coregulated Pilus (TCP) and the Chitin-regulated Competence Pilus (ChiRP) being important for biofilm formation on chitin [64, 69, 70]. However, understanding the precise mechanisms underlying *V. cholerae* biofilm formation on chitin and why they evolved a mechanistically distinct way of growing on chitin versus other surfaces remains an interesting and important area for future work.

Another overarching observation from this work is that *B. bacteriovorus* abundance on and within *V. cholerae* biofilms is dependent on production of VPS. The observation that the VPS-dependent matrix both contributes to prey protection from predator exposure in the biofilm interior while also retaining some *B. bacteriovorus* cells in place suggests the possibility that biofilm architecture can stabilize predator–prey dynamics. This type of predator–prey interaction pattern may, in turn, contribute to the observed trophic diversity of naturally occurring biofilms [11, 76–80]. Even though the mechanism by which VPS-dependent biofilm architecture affects *B. bacteriovorus* abundance in the biofilm interior is unknown, we speculate that VPS and matrix protein production lead to biofilm architectures that reduce overall prey accessibility for *B. bacteriovorus* while also permitting some degree of entanglement that holds the predatory species in areas of the biofilm where matrix maturation has not yet occurred completely. Understanding the molecular mechanisms of this proposed interaction will be key to a fuller understanding of how biofilm matrix physiology influences microbial predator–prey dynamics more broadly.

## Supplementary Material

Supplementary_material_wrag072

## Data Availability

All data generated or analyzed during this study are included in this published article.
