# Supplementary material for: Predator–prey dynamics of Vibrio cholerae on chitin suggest an alternative mode of biofilm formation in marine snow conditions
Source: ISME J. 2026 Mar 30;20(1):wrag072. doi: 10.1093/ismejo/wrag072 (PMC13157834; doi:10.1093/ismejo/wrag072)
Supplement: Supplementary_material_wrag072 [file supplementary_material_wrag072.zip › Holt_etal_ISMEJRevision2_SI_Update2.pdf]

Supporting Information

Predator-prey dynamics of *Vibrio cholerae* on chitin suggest an alternative mode of biofilm formation in marine snow conditions

Jacob D. Holt[1,2], Katherine A. Miller[1], Olivia F. Hunter[1], Emily Zhang[3], Alexander J. Hinbest[4], Emma Gerace[4], Rich Olson[4], Daniel E. Kadouri[3], and Carey D. Nadell[1,2]*

1. Department of Biological Sciences, Dartmouth, Hanover, New Hampshire, USA
2. Department of Microbiology and Immunology, Geisel School of Medicine at Dartmouth, Hanover, New Hampshire, USA
3. Department of Oral Biology, Rutgers School of Dental Medicine, Newark, NJ, USA
4. Department of Molecular Biology and Biochemistry, Molecular Biophysics Program, Wesleyan University, Middletown, CT, USA

Correspondence:

Carey D. Nadell
78 College Street
Hanover, NH 03755
carey.d.nadell@dartmouth.edu

Author Contributions: CDN funded and supervised the study. JDH and CDN conceived the study. JDH, CDN, and DK designed experiments. JDH, KM, and OH performed experiments. JDH constructed strains, performed modeling, analyzed data, and generated the figures. EZ, AH, EM, RO, and DK provided key software, reagents, and strains. JDH and CDN wrote the paper. All authors contributed to manuscript editing.

SI Figures

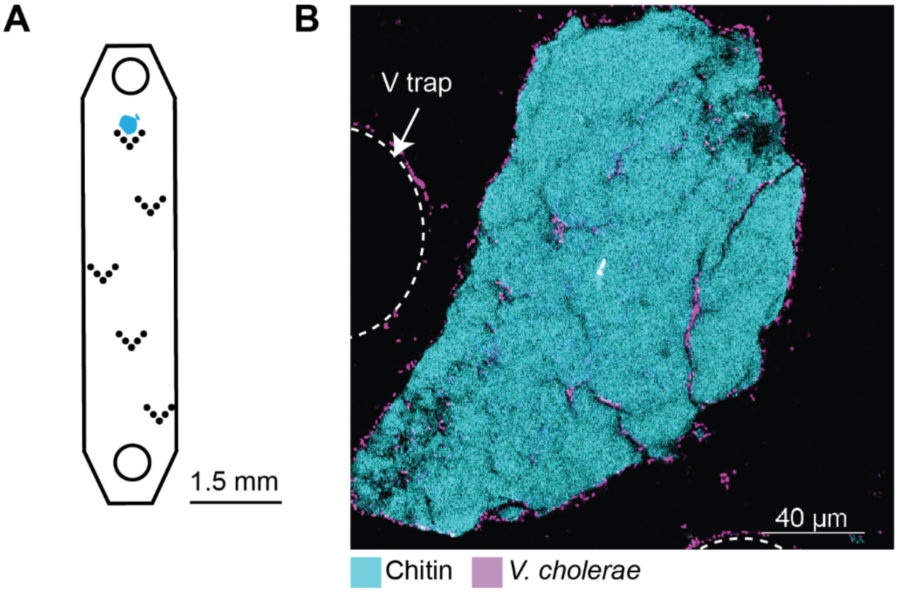

SI Figure S1: V trap microfluidic device. **(A)** Cartoon illustration of the V trap microfluidic device chamber design, drawn to scale. **(B)** Representative image of a *V. cholerae*-colonized chitin flake wedged between two columns of a V trap. Chitin is shown in cyan, *V. cholerae* is shown in purple, and the V trap columns visible in this field of view are shown as white, dashed lines (at left, and bottom-right).

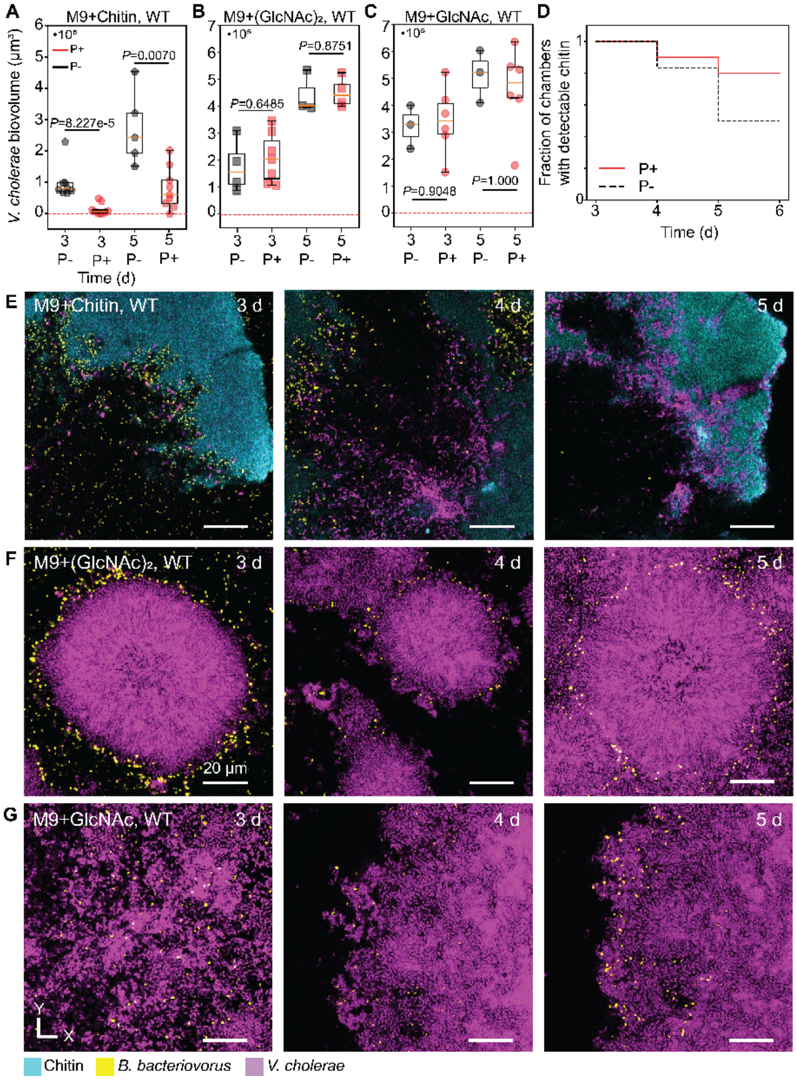

SI Figure S2: *V. cholerae* biofilms grown on chitin have increased susceptibility to predation relative to biofilms grown in GlcNAc or $(GlcNAc)_2$. **(A)** Box and whisker plot comparing predation to no predation in biofilms grown on chitin at 3 d (~4 h post introduction of predator) and 5 d, illustrating a significant decrease in *V. cholerae* biofilm abundance (Mann-Whitney *U* test, n=5-9). **(B)** Box and whisker plot comparing predation to no predation in biofilms grown on glass with $(GlcNAc)_2$ at 3 d (~4 h post introduction of predator) and 5 d, illustrating no significant difference in *V. cholerae* biofilm abundance (Mann-Whitney *U* test, n=3-7). **(C)** Box and whisker plot comparing the predation and no-predation conditions for biofilms grown on glass with GlcNAc at 3 d (~4 h post introduction of predator) and 5 d, illustrating no significant decrease in *V. cholerae* biofilm abundance (Mann-Whitney *U* test, n=3-6). **(D)** Fraction of microfluidic chambers with detectable chitin plotted against time for the *V. cholerae* biofilms that were invaded by *B. bacteriovorus*, shown as the red line, and biofilms that were not invaded, shown as the dashed black line. **(E)** Representative images of *V. cholerae* biofilms being predated by *B. bacteriovorus* when grown with chitin particles as the sole carbon source. **(F)** Representative images of *V. cholerae* biofilms being predated by *B. bacteriovorus* when grown on glass with soluble $(GlcNAc)_2$ as the sole carbon source. **(G)** Representative images of *V. cholerae* biofilms being predated by *B. bacteriovorus* when grown on glass with soluble GlcNAc as the sole carbon source. (E-F) *V. cholerae* is shown in purple, *B. bacteriovorus* is shown in yellow, and chitin is shown in cyan.

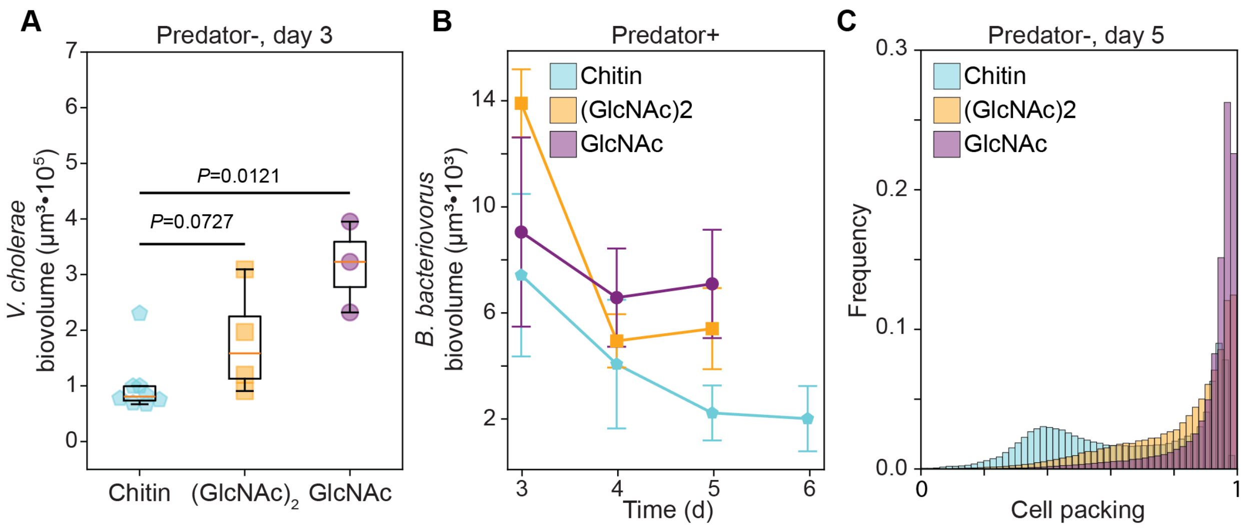

SI Figure S3: (A) *V. cholerae* abundance at day 3 without predator exposure, illustrating a significant difference in biovolume accumulation between the chitin and glass with soluble GlcNAc growth conditions (Mann-Whitney *U* test, n=3-8) **(B)** Population dynamics of *B. bacteriovorus* over time following invasion into *V. cholerae* biofilms formed on chitin (cyan trace) or on glass with GlcNAc (purple trace), or $(GlcNAc)_2$ (orange trace) as the sole carbon source. **(C)** *V. cholerae* cell packing frequency distributions after 5 d of growth in each of the three biofilm growth conditions.

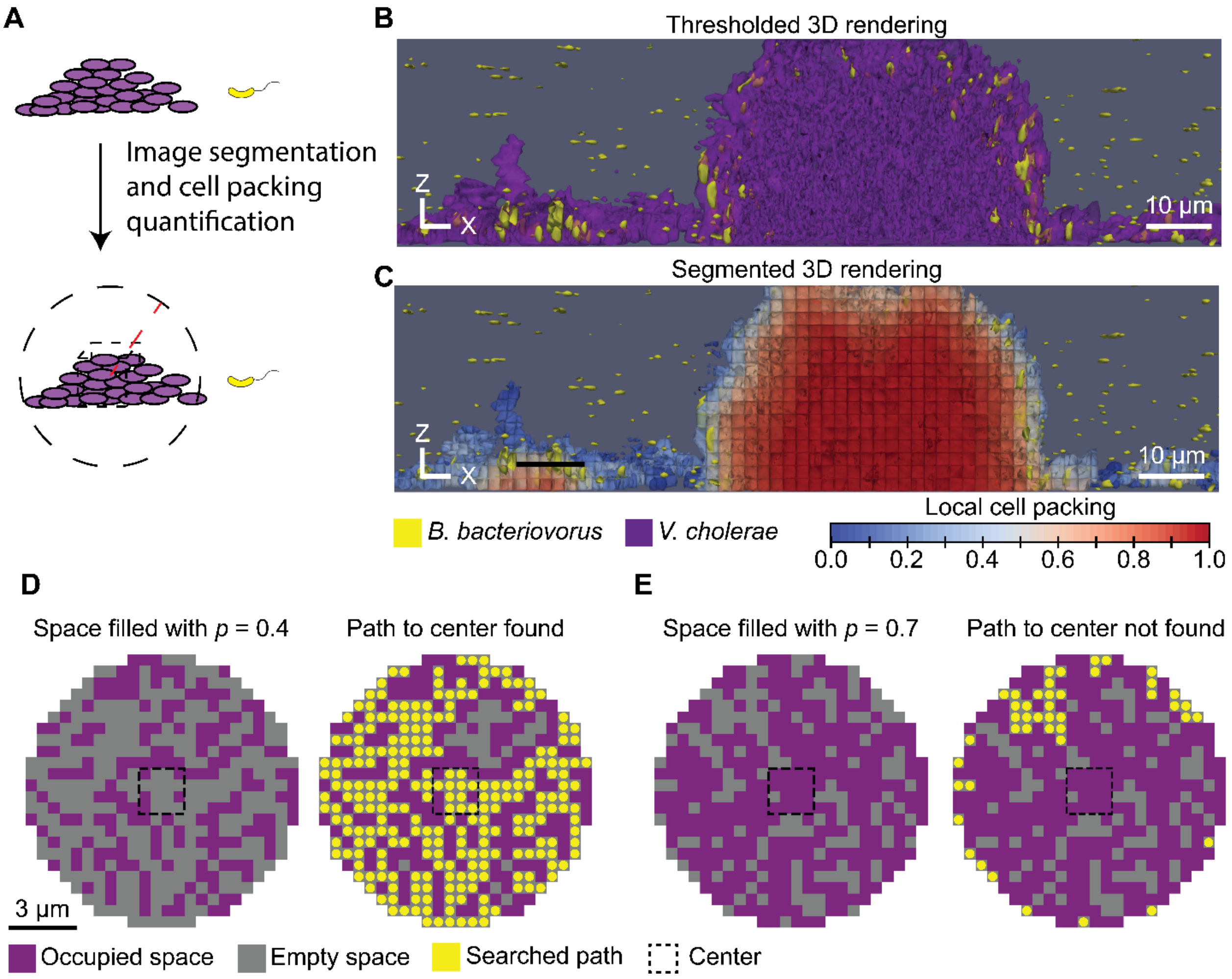

SI Figure S4: Percolation model schematic. **(A)** Diagram detailing how BiofilmQ calculates local cell packing. For each segmented *V. cholerae* biovolume, the fraction of space within a volume of fixed radius from the centroid of the segmented volume is measured to obtain a biovolume fraction, which we term cell packing. *V. cholerae* is shown in purple, *B. bacteriovorus* is shown in yellow, the BiofilmQ radius parameter is shown as a dashed red line, the volume over which BiofilmQ calculates local cell packing fraction is shown as a black dashed line, and the BiofilmQ pseudo-cell cube-object is shown as a cube. **(B)** A side profile representative 3D rendering of a thresholded *V. cholerae* biofilm being predated upon by *B. bacteriovorus*. *V. cholerae* is shown in purple and *B. bacteriovorus* is shown in yellow. **(C)** A side profile representative 3D rendering of a segmented and quantified *V. cholerae* biofilm being predated upon by *B. bacteriovorus*. *V. cholerae* segmented biovolume is colored by the cell packing measurement and *B. bacteriovorus* is shown in yellow. (**D)** Representative simulation of percolation on a 2D circle with a cell packing fraction of 0.4. **(E)** Representative 2D simulation of percolation with a cell packing fraction of 0.7. Occupied space is shown in purple, open space is shown in grey, the searched path is shown in yellow, and the center, representing segmented biovolume, is shown as a dashed square. 2D simulations are shown for visualization purposes, while the quantified simulation data shown in Figures 1 and 2 comes from 3D (spherical) simulations.

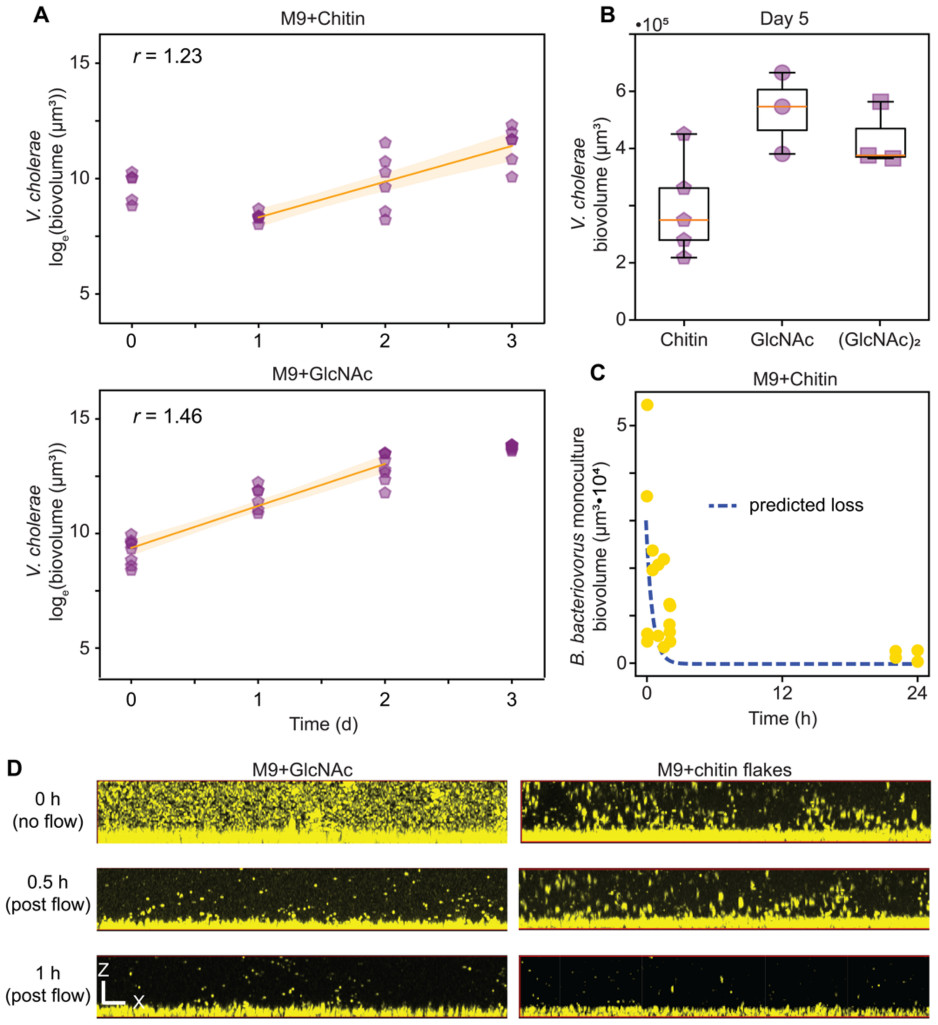

SI Figure S5: Monoculture data used to parameterize the Lotka-Volterra model. **(A)** Plot of natural log of biovolume against time for WT *V. cholerae* grown in M9 with chitin flakes or M9 with GlcNAc as the sole carbon source. Solid orange line represents the line of best fit used to experimentally determine the biofilm maximal growth rate, *r*. **(B)** Box and whisker plot of WT *V. cholerae* biofilm volume at day 5, used to determine the biofilm carrying capacity, *K*. **(C)** Population dynamics of *B. bacteriovorus* over 24 h in microfluidic devices not containing prey biofilm. The dashed blue line is the microfluidic device dilution rate, which was used to set the *B. bacteriovorus* loss rate from the biofilm, *d*. **(D)** Representative x-z maximal intensity projection series of *B. bacteriovorus* monoculture in M9+Chitin (right) and in M9 with GlcNAc (left). *B. bacteriovorus* is shown in yellow.

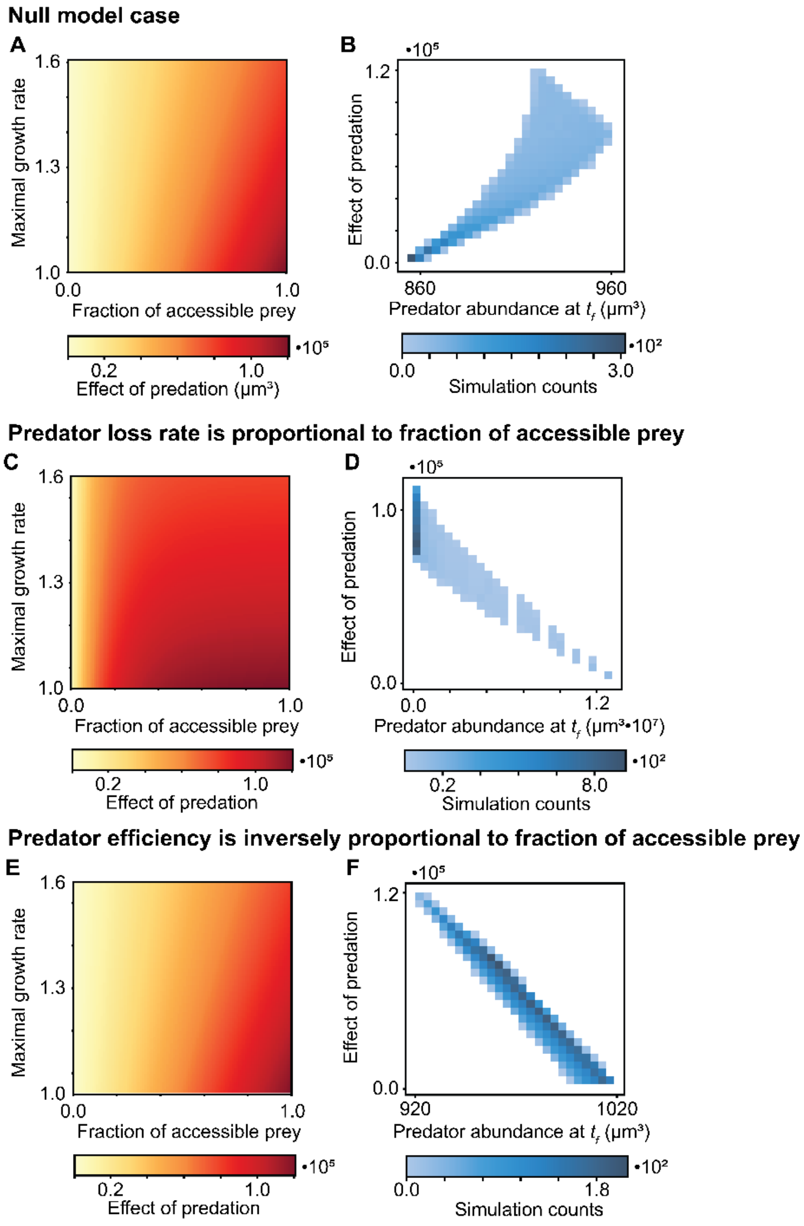

SI Figure S6: Response of predator abundance as model assumptions are altered. **(A)** Plot of the predator's effect on its prey as the fraction of accessible prey and the maximal growth rate are varied. Replot of the simulation results from Figure 1I. **(B)** 2D histogram of predator abundance at the simulation end (6 d) against effect of predation for all model runs showing that an increase in accessible prey correlates positively with predator abundance. **(C)** Plot of the predator's effect on its prey as the fraction of accessible prey and the maximal growth rate are varied in the model case where the predator's loss rate is modified proportionally to the fraction of accessible prey. **(D)** 2D histogram of predator abundance at the simulation end (6 d) against effect of predation for all model runs showing that an increase in accessible prey correlates positively with predator abundance. **(E)** Plot of the predator's effect on its prey as the fraction of accessible prey and the maximal growth rate are varied in the model case where the predator's efficiency is modified inversely to the fraction of accessible prey. **(F)** 2D histogram of predator abundance at the simulation end (6 d) against effect of predation for all model runs showing that an increase in accessible prey correlates positively with predator abundance.

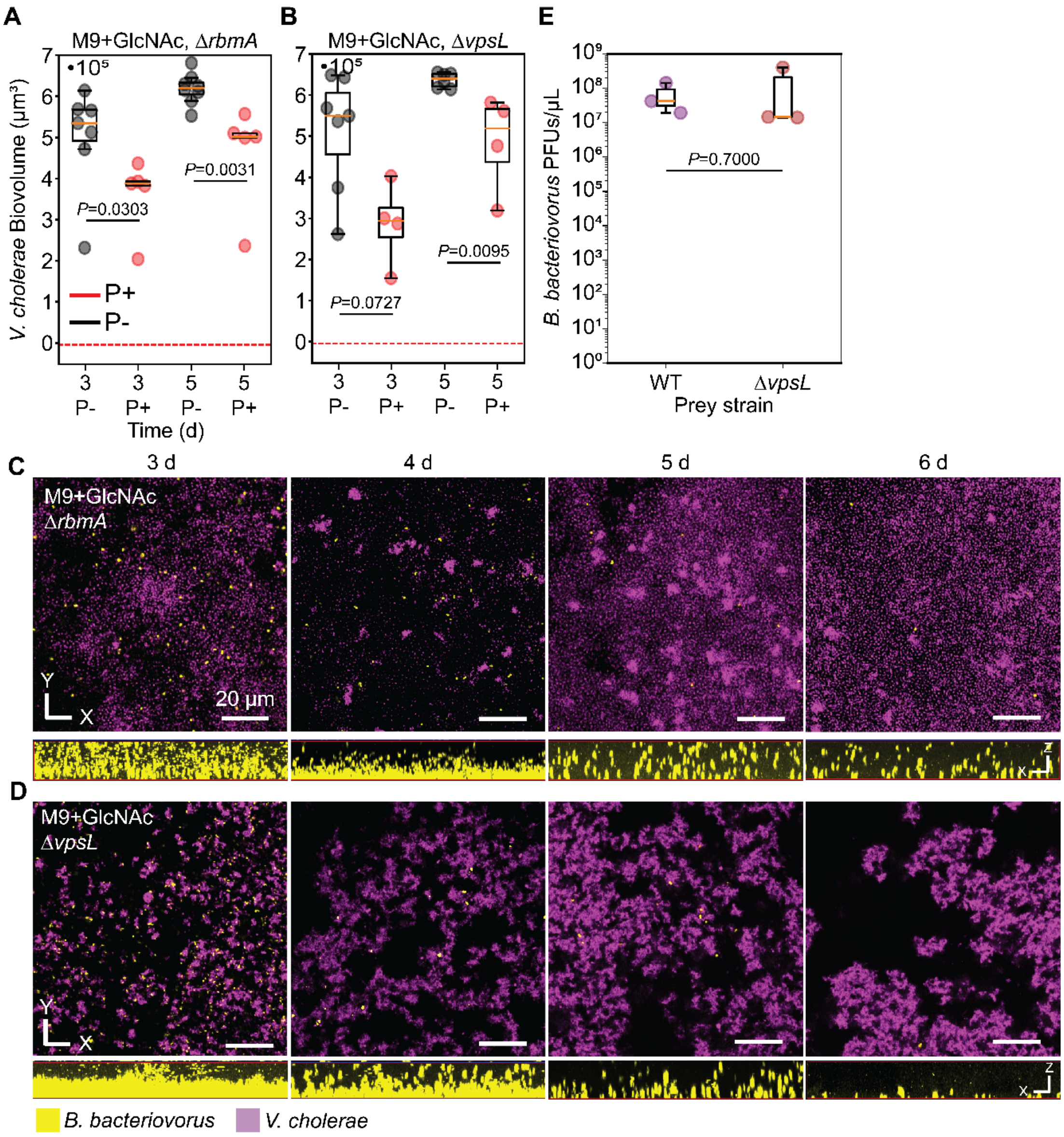

SI Figure S7: Dense local cell packing is necessary for biofilm level protection against *B. bacteriovorus,* and VPS secretion supports *B. bacteriovorus* maintenance. **(A)** Box and whisker plot comparing predation to no predation Δ*rbmA* biofilms grown in GlcNAc at 3 d (~4 h post introduction of predator) and 5 d showing a significant decrease in *V. cholerae* biofilm abundance (Mann-Whitney *U* test, n=4-7). **(B)** Box and whisker plot comparing predation to no predation Δ*vpsL* biofilms grown in GlcNAc at 3 d (~4 h post introduction of predator) and 5 d showing a significant difference in *V. cholerae* biofilm volume (Mann-Whitney *U* test, n=4-7). **(C)** Representative images of *V. cholerae* Δ*rbmA* biofilms being predated upon by *B. bacteriovorus* over time with x-z maximum intensity projections of *B. bacteriovorus* shown below. **(D)** Representative images of *V. cholerae* Δ*vpsL* biofilms being predated upon by *B. bacteriovorus* over time with x-z maximum intensity projections of *B. bacteriovorus* shown below. **(E)** *B. bacteriovorus* plaques with equal efficiency on WT and Δ*vpsL* prey cells (Mann-Whitney *U* test, n=3)**.** *V. cholerae* is shown in purple and *B. bacteriovorus* is shown in yellow.

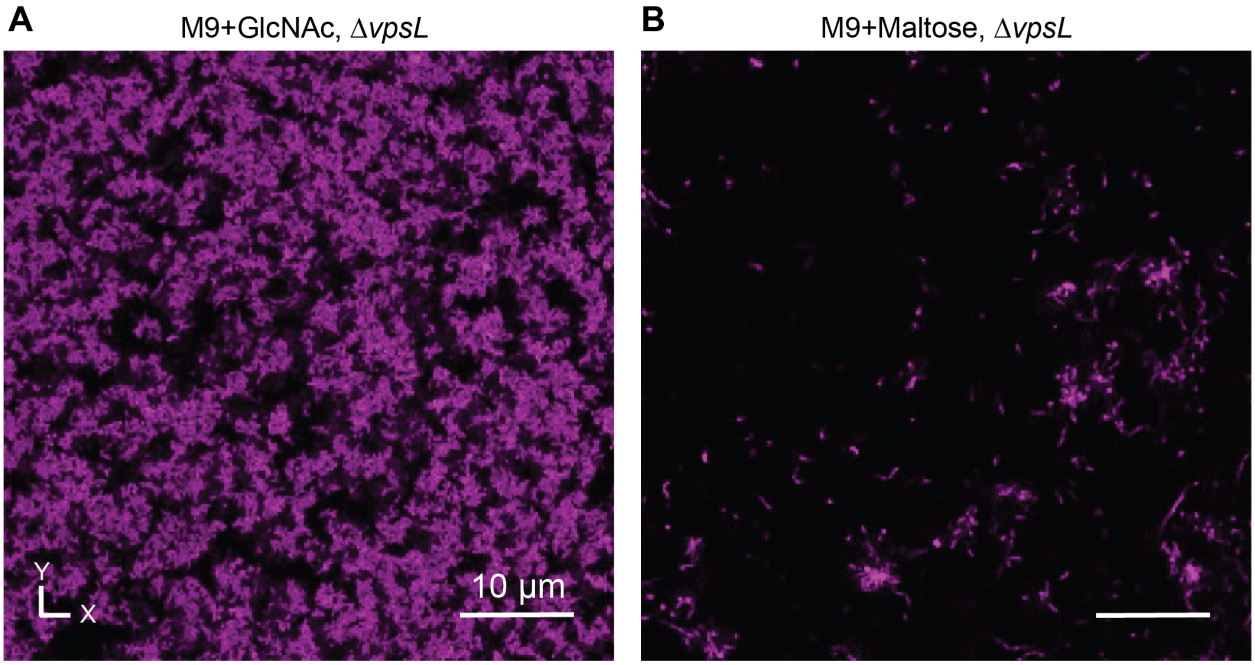

SI Figure S8: *V. cholerae* Δ*vpsL* forms disordered 3-dimensional biofilms dependent on media conditions. **(A)** *V. cholerae* Δ*vpsL* forms 3-dimensional biofilms when grown with GlcNAc as the sole carbon source. **(B)** *V. cholerae* Δ*vpsL* does not form 3-dimensional biofilms when grown with maltose as the sole carbon source. *V. cholerae* Δ*vpsL* is shown in purple.

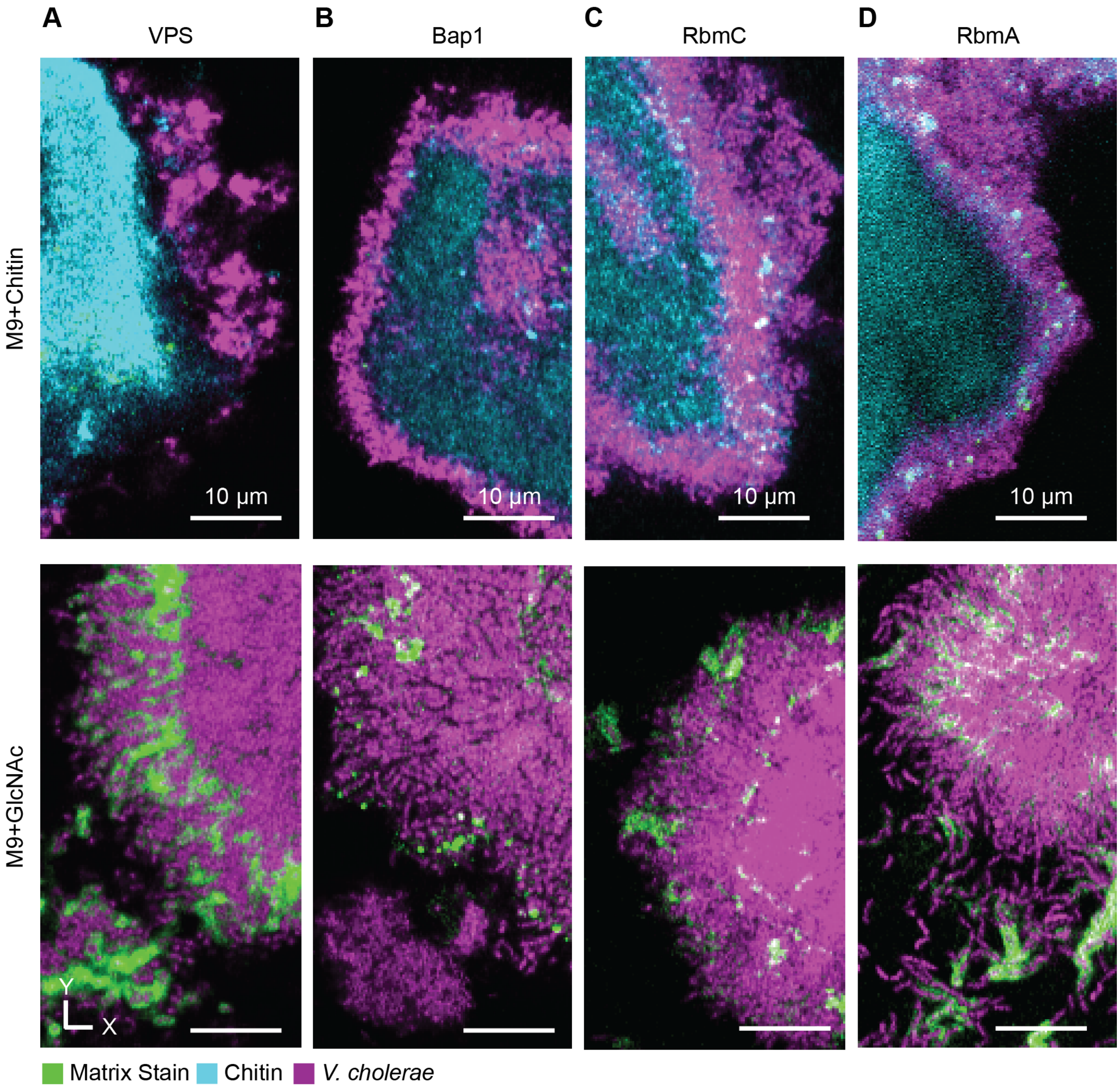

SI Figure S9: *V. cholerae* biofilms formed on chitin contain less *Vibrio* exopolysaccharide, RbmA, RbmC, and Bap1 relative to biofilms formed in GlcNAc. **(A)** Representative image of *V. cholerae* grown on chitin (top) stained for VPS alongside a representative image of *V. cholerae* grown in GlcNAc also stained for VPS (bottom). **(B)** Representative image of *V. cholerae* grown on chitin (top) stained for Bap1 alongside a representative image of *V. cholerae* grown in GlcNAc also stained for Bap1 (bottom). **(C)** Representative image of *V. cholerae* grown on chitin (top) stained for RbmC alongside a representative image of *V. cholerae* grown in GlcNAc also stained for RbmC (bottom). **(D)** Representative image of *V. cholerae* grown on chitin (top) stained for RbmA alongside a representative image of *V. cholerae* grown in GlcNAc also stained for RbmA (bottom). In all image panels matrix stain is shown in green, chitin is shown in cyan, and *V. cholerae* is shown in purple.

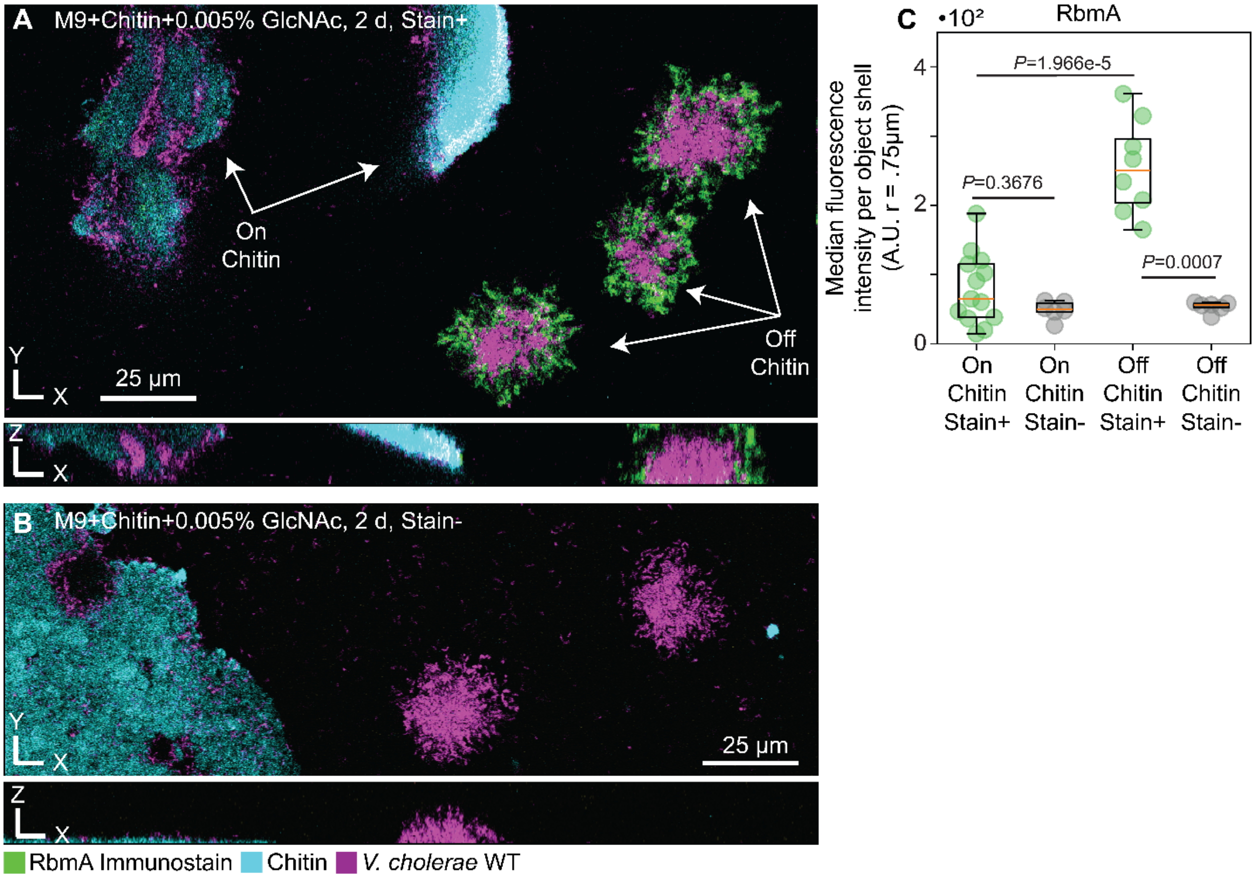

SI Figure S10: Biofilms formed in microfluidic devices with chitin and GlcNAc in the influent medium stain for RbmA differently depending on the surface to which they are attached. **(A)** Representative image of RbmA immunostaining from a dual carbon source experiment in which *V. cholerae* was cultivated with chitin flakes, GlcNAc at 0.005%, and RbmA immunostain in the influent medium. **(B)** Representative image of RbmA immunostain signal from a control experiment where RbmA immunostain was not present in the media. *V. cholerae* is shown in purple, chitin is shown in cyan, and RbmA immunostain signal is shown in green. **(C)** Immunostaining of FLAG tagged RbmA quantified as the median pseudo-cell cube fluorescence intensity within a 0.75 µm shell is significantly less on chitin relative to cell groups adhered to glass in the dual carbon course condition (Mann-Whitney *U* test, n=8-13 regions) and insignificantly different from the control lacking matrix stain (Mann-Whitney *U* test, n=6-13 regions). The microcolonies formed off chitin displayed significantly higher matrix stain fluorescence intensity compared to the control where matrix stain was not added (Mann-Whitney *U* test, n=6-8 regions).

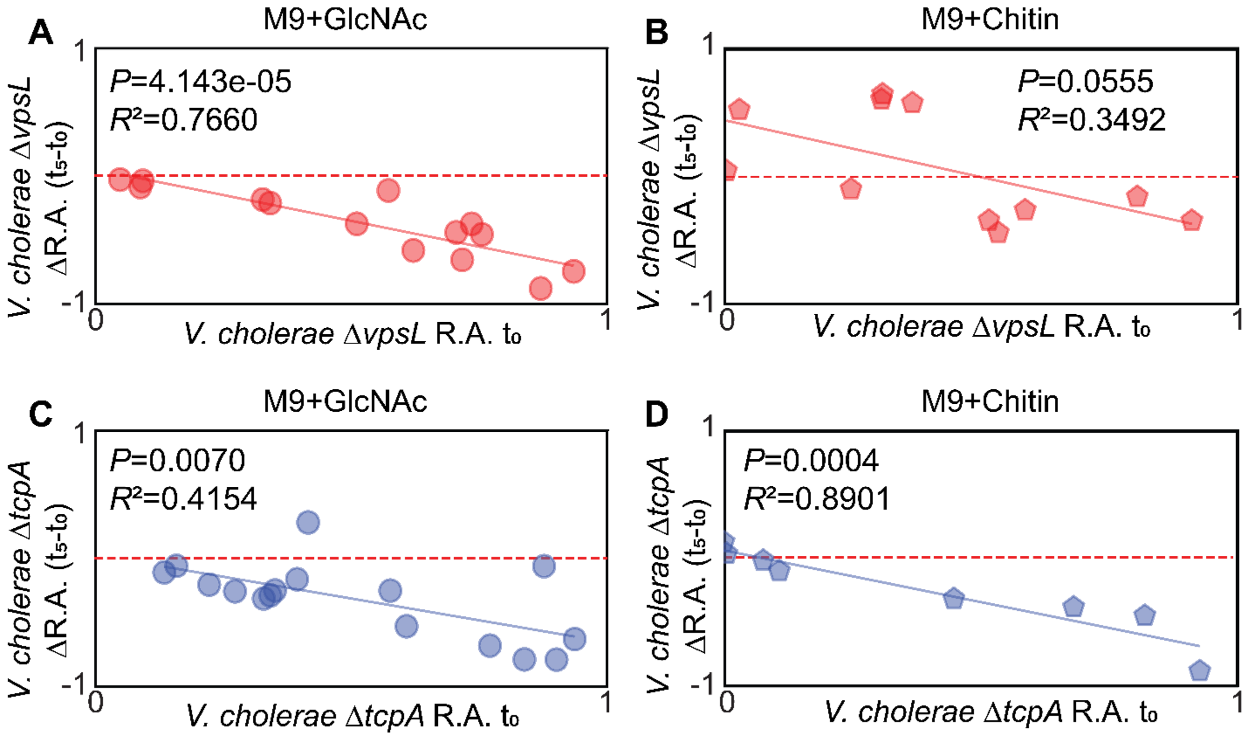

SI Figure S11: Growth substrate alters the competitive ability of WT against Δ*vpsL* and Δ*tcpA* **(A)** Plot of *V. cholerae* Δ*vpsL* initial relative abundance against the change in *V. cholerae* Δ*vpsL* relative abundance after five days of coculture with WT grown in M9 with GlcNAc as the sole carbon source. **(B)** Plot of *V. cholerae* Δ*vpsL* initial relative abundance against the change in *V. cholerae* Δ*vpsL* relative abundance after five days of coculture with WT grown in M9 with Chitin as the sole carbon source. **(C)** Plot of *V. cholerae* Δ*tcpA* initial relative abundance against the change in *V. cholerae* Δ*tcpA* relative abundance after five days of coculture with WT grown in M9 with GlcNAc as the sole carbon source. **(D)** Plot of *V. cholerae* Δ*tcpA* initial relative abundance against the change in *V. cholerae* Δ*tcpA* relative abundance after five days of coculture with WT grown in M9 with chitin as the sole carbon source. The red dashed line shows the zero-change line.

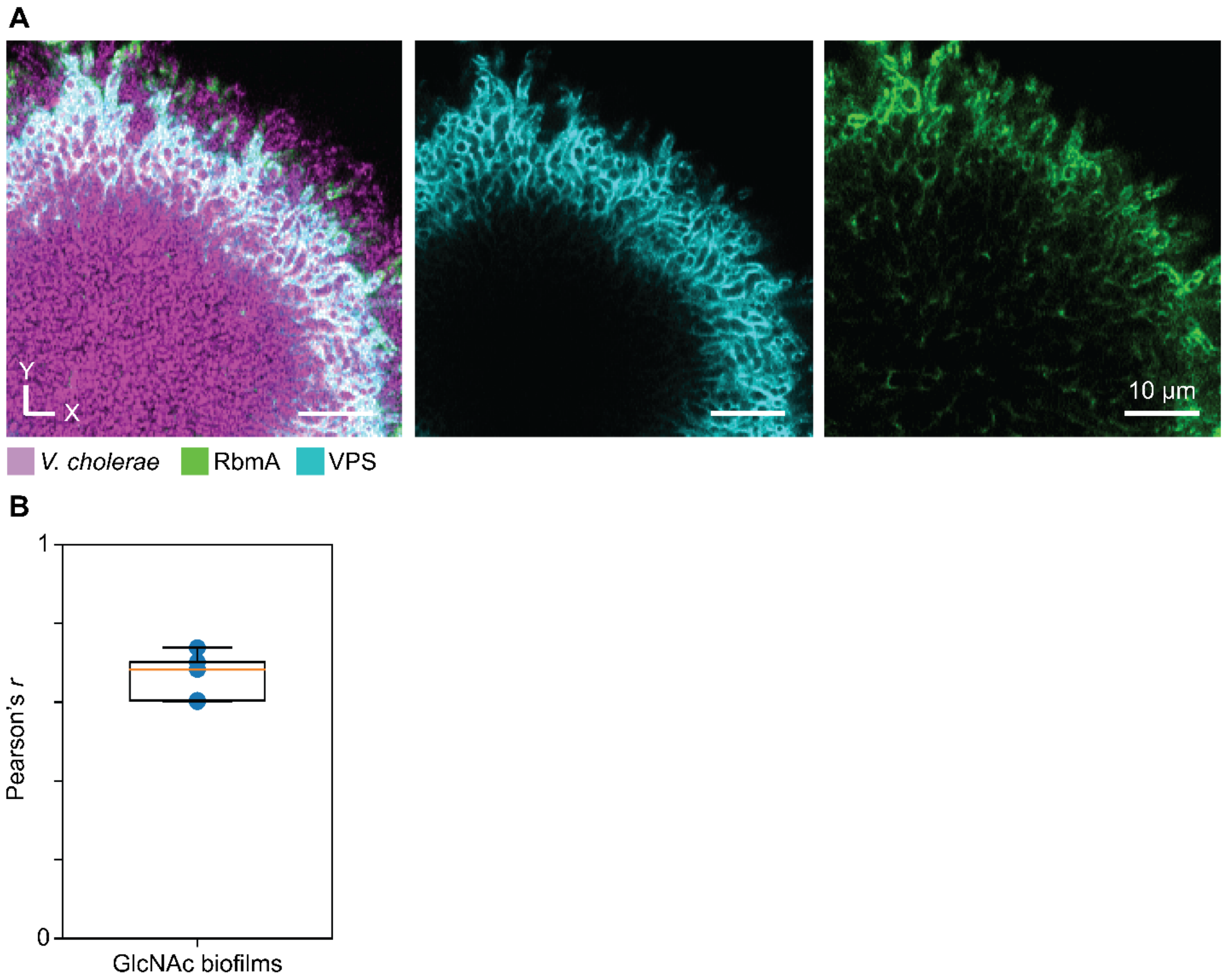

SI Figure S12: VPS stain is strongly correlated with RbmA stain. **(A)** Representative image of a V. *cholerae* biofilm grown in GlcNAc exposed continuously to RbmA immunostain for 2 d and then stained with a spike in of Bap1 bound to Alexa Fluor 488. *V. cholerae* is shown in purple, VPS stain is shown in cyan, and RbmA stain is shown in green. **(B)** Pearson's correlation coefficient of VPS and RbmA stain signal (n = 5 images).

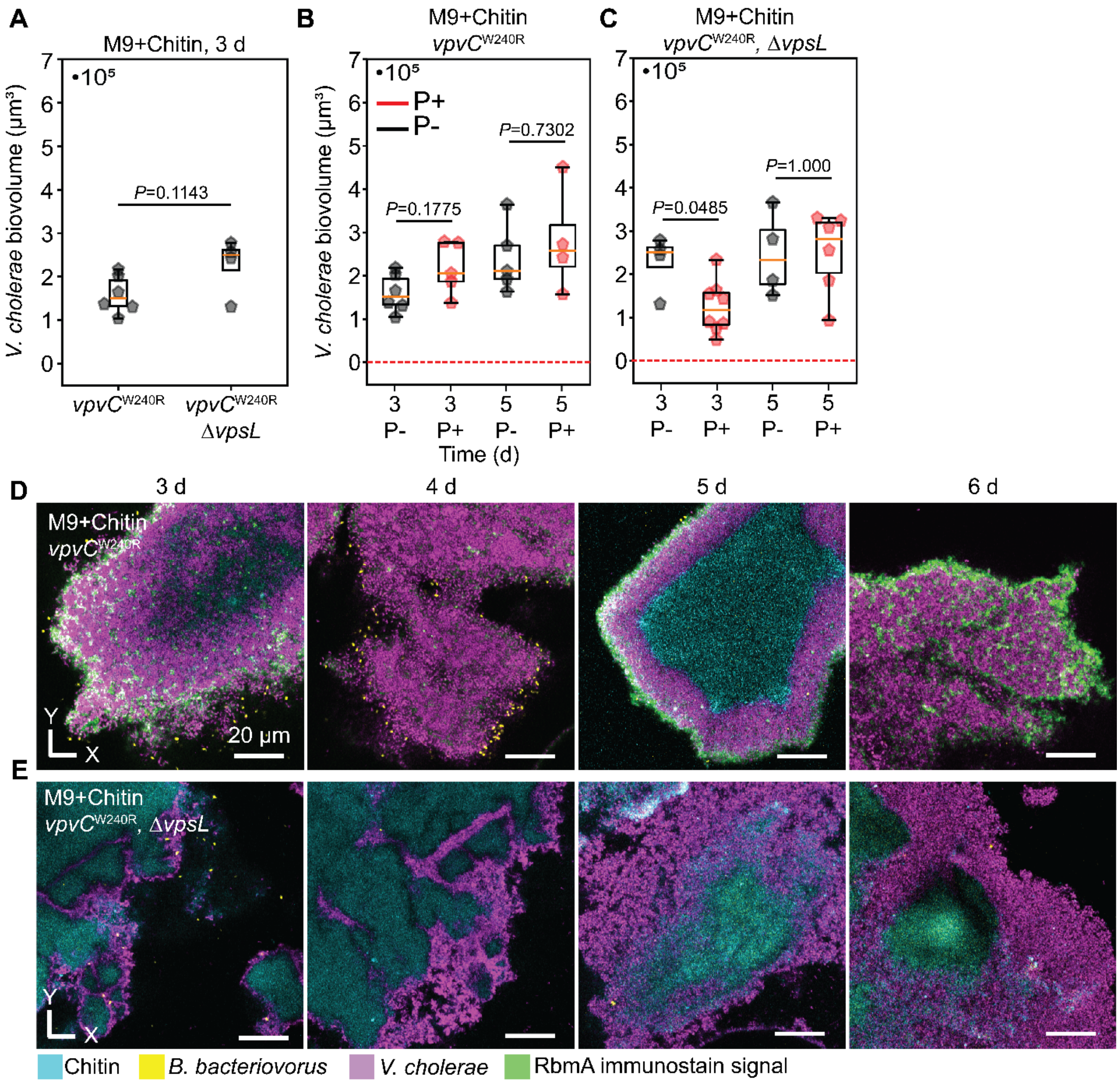

SI Figure S13: *V. cholerae vpvC*^{W240R} biofilms are protected from predation in a VPS-dependent manner when grown on chitin. **(A)** Direct comparison of *vpvC*^{W240R} and *vpvC*^{W240R} Δ*vpsL* biofilm biovolume at day 3 showing no significant difference (Mann-Whitney *U* test, n=4-6). **(B)** Box and whisker plot comparing predation to no predation *vpvC*^{W240R} biofilms grown on chitin at 3 d (~4 h post introduction of predator) and 5 d showing no significant difference in *V. cholerae* biofilm volume (Mann-Whitney *U* test, n=4-6). **(C)** Box and whisker plot comparing predation to no predation *vpvC*^{W240R} Δ*vpsL* biofilms at 3 d (~4 h post introduction of predator) and 5 d showing a significant difference (Mann-Whitney *U* test, n=4-6). **(D)** Representative images of *V. cholerae vpvC*^{W240R} biofilms being predated upon by *B. bacteriovorus* over time. **(E)** Representative images of *V. cholerae vpvC*^{W240R} Δ*vpsL* biofilms being predated upon by *B. bacteriovorus* over time. *V. cholerae* is shown in purple, *B. bacteriovorus* is shown in yellow, chitin is shown in cyan, and RbmA immunostaining signal is shown in green.

SI Tables

SI Table S1: List of strains, materials, and software.

**Bacterial Strains**	**Relevant markers/Genotype**	**Source**
V. cholerae		
CNV116	*rbmA*-3xFLAG, *lacZ*:Ptac-mKate2	PMID: 31239347 [1]
CNV119	*bap1*-3xFLAG, *lacZ*:Ptac-mKate2	PMID: 36730197 [2]
CNV117	*rbmC*-3xFLAG, *lacZ*:Ptac-mKate2	PMID: 36730197 [2]
CNV68	*ΔvpsL*, *vpvC-W240R*, *lacZ*:Ptac-mKate2	PMID: 24332540 [3]
CNV252	*vpvC-W240R*, *lacZ*:Ptac-mKate2, rbmA-3xFLAG	PMID: 33826904 [4]
CNV245	*ΔrbmA*, *lacZ*:Ptac-mKate2	PMID: 33826904 [4]
CNV126	*ΔrbmA*, *lacZ*:Ptac-mKo	PMID: 31659297 [5]
CNV52	*lacZ*:Ptac-mKate2	PMID: 24332540 [3]
CNV59	*ΔvpsL*, *lacZ*:Ptac-mKo	PMID: 24332540 [3]
CNV60	*ΔvpsL*, *lacZ*:Ptac-mKate2	PMID: 24332540 [3]
CNV121	*rbmA*-3xFLAG, *lacZ*:Ptac-mKo	PMID: 31239347 [1]
CNV333	*rbmA*-3xFLAG, *lacZ*:Ptac-mKate2, Δ*tcpA*	This study.
B. bacteriovorus		
109J	PMQ581, gfp-mut	PMID: 33826904 [4]
E. coli		
CNE793	PBW, Δ*tcpA*, AmpR, KanR, sacB	PMID: 31239347 [1]
**DNA Oligos**	**Sequence**	**Description**
CNO1044	ccacaaggtaacgtagtcaaaagttgga	Used to confirm *tcpA* deletion
CNO1045	ccgtatcccaacacaactcagtatcttc	Used to confirm *tcpA* deletion
**Chemicals and Reagents**	**Source**	**Product number**
DyLight 488 NHS-Ester	Thermo Fisher Scientific	cat. #46403
Poly-dimethysiloxane (PDMS)	Dow Chemical Company SYLGARD 184	cat. #04019862
#1.5 glass coverslips	Azer Scientific	cat. #1152260
Inlet tubing	Cole Palmer	cat. #06417-11

27Gx1/2 needles	BD Precision	cat. #30510
1mL syringes	Brandzig	cat. #CMD2583
Harvard Apparatus Pico Plus Elite syringe pumps	Harvard Apparatus	cat. #70-4506
Chitin flakes	Sigma-Aldrich	cat. #C9752
Chitobiose $(GlcNAc)_2$	BIOSYNTH	cat. #OD00769
N-Acetyl-D-glucosamine GlcNAc	Sigma-Aldrich	cat. #A8625
Glucose	Sigma-Aldrich	cat. #G7528
Maltose	Sigma-Aldrich	cat. #M5885
M9, Minimal Salts 5x	Sigma-Aldrich	cat. #M6030
Calcium Chloride	Sigma-Aldrich	cat. #C8106
Magnesium Sulfate	Sigma-Aldrich	cat. #M2643
MEM Vitamin Solution (100x)	Sigma-Aldrich	cat. #M6895
Monoclonal ANTI-FLAG M2-Cy3 antibody	Sigma-Aldrich	cat. #A9594
Lysogeny Broth (LB), Miller	Sigma-Aldrich	cat. #L3552
LB Agar, Miller	Sigma-Aldrich	cat. #L3147
Agar Powder	Thermo Scientific	cat. #A10752.36
Tryptone	Sigma-Aldrich	cat. #T7293
Yeast Extract	Sigma-Aldrich	cat. #70161
Kanamycin	Sigma-Aldrich	cat. #K1377

Polymyxin B	Sigma-Aldrich	cat. #P4932
Magnesium Chloride	Sigma-Aldrich	cat. #M4880
Nutrient Broth	Sigma-Aldrich	cat. #70122
HEPES Buffer	Fisher Scientific	cat. BP299
Sucrose	Sigma-Aldrich	cat. #S0389
**Software and Algorithms**	**Source**	**Version**
Zen Black	Zeiss	v14.0.0.0
Zen Blue	Zeiss	v3.4.91.00000
MATLAB	MathWorks [6]	vR2021a
BiofilmQ	PMID:33398098 [7]	v0.2.2
Python	Python.org [8]	v3.8.8
Anaconda	Anaconda.org	v2021.05
Spyder	Spyder-ide.org	v4.2.5
NumPy	NumPy.org [9]	v1.20.1
Matplotlib	Matplotlib.org [10]	v3.3.4
seaborn	Seaborn.pydata.org [11]	v0.11.1
SciPy	Scipy.org [12]	v1.6.2
Pandas	Pandas.pydata.org [13]	v1.2.4
Paraview	Kitware	v9.4.1

SI Table S2: List of percolation model parameters.

Parameter	Symbol	Value	Unit
Cubic center	*C*	4	Voxels ($0.5\ \mu m^3$)
Radius	*r*	12	Voxels ($0.5\ \mu m^3$)
Samples	*N*	400	unitless

SI Table S3: List of ODE model parameters.

Parameter	Symbol	Value	Unit
V. cholerae maximal growth rate	*r*	0.5-1.5 (SI Figure S4)	d^{-1}
V. cholerae carrying capacity	*K*	$3 \cdot 10^5$-$5 \cdot 10^5$ (SI Figure S4)	μm^3
B. bacteriovorus attack rate	*a*	$5.52 \cdot 10^{-7}$ [14]	$\mu m^{-3}\ d^{-1}$
B. bacteriovorus loss rate	*d*	1.7952 (SI Figure S4)	d^{-1}
Bdelloplast maturation rate	k_p	2.616 [15]	d^{-1}
Bdelloplast to predator conversion factor	*b*	0.438 [15]	unitless
Predator-prey interaction term	*v*	0-1 (Figure 1, SI Figure S5)	unitless

References:

1. Wucher BR, Bartlett TM, Hoyos M, et al. *Vibrio cholerae* filamentation promotes chitin surface attachment at the expense of competition in biofilms. *Proc. Natl. Acad. Sci. USA* 2019;**116**:14216–14221. https://doi.org/10.1073/pnas.1819016116
2. Wucher BR, Winans JB, Elsayed M, et al. Breakdown of clonal cooperative architecture in multispecies biofilms and the spatial ecology of predation. *Proc. Natl. Acad. Sci. USA* 2023;**120**:e2212650120. https://doi.org/10.1073/pnas.2212650120
3. Drescher K, Nadell CD, Stone HA, et al. Solutions to the public goods dilemma in bacterial biofilms. *Curr. Biol.* 2014;**24**:50–55. https://doi.org/10.1016/j.cub.2013.10.030
4. Wucher BR, Elsayed M, Adelman JS, et al. Bacterial predation transforms the landscape and community assembly of biofilms. *Curr. Biol.* 2021;**31**:2643-2651.e3. https://doi.org/10.1016/j.cub.2021.03.036
5. Díaz-Pascual F, Hartmann R, Lempp M, et al. Breakdown of *Vibrio cholerae* biofilm architecture induced by antibiotics disrupts community barrier function. *Nat. Microbiol.* 2019;**4**:2136–2145. https://doi.org/10.1038/s41564-019-0579-2
6. Inc TM. MATLAB version: 9.13.0 (R2021a). 2022. Natick, Massachusetts, United States: The MathWorks Inc., 2022.
7. Hartmann R, Jeckel H, Jelli E, et al. Quantitative image analysis of microbial communities with BiofilmQ. *Nat. Microbiol.* 2021;**6**:151–156. https://doi.org/10.1038/s41564-020-00817-4
8. Pilgrim M, Willison S. Dive Into Python 3. Springer, 2009.
9. McKinney W. Data structures for statistical computing in Python. In: Walt S van der, Millman J (eds) *Proceedings of the 9th Python in Science Conference*. 2010. 2010, pp 56–61.
10. Hunter JD. Matplotlib: A 2D Graphics Environment. *Comput. Sci. Eng.* 2007;**9**:90–95. https://doi.org/10.1109/MCSE.2007.55

11. Waskom M. Seaborn: statistical data visualization. *JOSS* 2021;**6**:3021. https://doi.org/10.21105/joss.03021

12. SciPy 1.0 Contributors, Virtanen P, Gommers R, et al. SciPy 1.0: fundamental algorithms for scientific computing in Python. *Nat. Methods.* 2020;**17**:261–272. https://doi.org/10.1038/s41592-019-0686-2

13. team T pandas development. pandas-dev/pandas: Pandas. 2020. Zenodo, 2020.

14. Hobley L, Summers JK, Till R, et al. Dual predation by bacteriophage and *Bdellovibrio bacteriovorus* can eradicate *Escherichia coli* Prey in situations where single predation cannot. *J Bacteriol* 2020;**202:** e00629-19. https://doi.org/10.1128/JB.00629-19

15. Summers JK, Kreft J-U. Predation strategies of the bacterium *Bdellovibrio bacteriovorus* result in overexploitation and bottlenecks. *Appl. Environ. Microbiol.* 2022;**88**:e01082-21. https://doi.org/10.1128/AEM.01082-21
